# Nontoxigenic *Bacteroides fragilis* as a Next-Generation Probiotic: Mechanisms, Safety, and Therapeutic Potential

**DOI:** 10.3390/microorganisms14081795

**Published:** 2026-08-14

**Authors:** Dong Wang, Zheng Nie, Wenzheng Zhang, Jinhui Liu, Changqi Ge, Yannan Zhang, Yabin Lu, Zhanhai Mai, Xiaodong He, Jianlong Li, Chao Gong, Qingyong Guo

**Affiliations:** 1 College of Veterinary Medicine, Xinjiang Agricultural University, Urumqi 830052, China; blackwhiteelegant@163.com (D.W.); 15136000676@163.com (Z.N.); 15293814767@163.com (W.Z.); 17870102312@163.com (J.L.); 19862247478@163.com (C.G.); zynmed@163.com (Y.Z.); lyb4095@163.com (Y.L.); mzh881231@126.com (Z.M.); shouyihexiaodong@sina.com (X.H.); pbb0108@163.com (J.L.); 18328042911@163.com (C.G.); 2 Xinjiang Key Laboratory of New Drug Research and Development for Herbivorous Animals, Urumqi 830052, China; 3 Xinjiang Regional Key Laboratory of Clinical Veterinary Medicine Research, Urumqi 830052, China; 4 Herbivorous Animal Biomedicine and Diagnostic Technology Engineering Research Center, Urumqi 830052, China

**Keywords:** *Bacteroides fragilis*, beneficial effect, immune regulation, intestinal barrier

## Abstract

Nontoxigenic *Bacteroides fragilis* (NTBF) is defined by the absence of the *bft* gene and corresponding *B. fragilis* toxin production; however, nontoxigenic status alone does not establish uniform safety or probiotic function. This review critically evaluates strain-level biological characteristics, safety, mechanisms, metabolites, and disease-model evidence. Selected strains and defined strain-derived preparations, including ZY-312, HCK-B3, NCTC 9343-derived polysaccharide A, and ZY-312-derived zwitterionic capsular polysaccharide preparation TP2, have shown immunomodulatory, barrier-associated, and microbial-community-modulating activities, predominantly in vitro and in animal models. Direct causal evidence is limited to specific strain–preparation–host–model combinations, whereas many changes in cytokines, tight-junction-associated proteins, microbial composition, and organic-acid profiles remain functional or associative. Protective effects have been reported in preclinical models of inflammatory bowel disease, necrotizing enterocolitis, antibiotic-associated diarrhea, *Clostridioides difficile* infection, and enterotoxigenic *B. fragilis* (ETBF)-associated tumorigenesis. However, findings are highly dependent on the strain, preparation, dose, administration timing, host, and model. Safety remains incompletely resolved because the absence of *B. fragilis* toxin (BFT) does not exclude opportunistic or systemic infection, antimicrobial-resistance mobility, bacterial translocation, permeability changes, or adverse effects during long-term administration. Metabolic effects may also be beneficial or adverse depending on the strain, host dietary and genetic background, and experimental context. Human evidence is primarily observational, and robust intervention trials are lacking. Accordingly, NTBF should be regarded as a heterogeneous group of strain-specific live-biotherapeutic candidates requiring rigorous strain-specific manufacturing, potency, dose, antimicrobial-susceptibility, and safety evaluation.

## 1. Introduction

Intestinal health is the foundation of an animal’s overall health, and the gut microbiota plays a regulatory role in intestinal health [1]. When intestinal homeostasis is disrupted, such as during diarrhea, the ion transport, physical barrier, and microbial balance in the gut are all impaired. This not only leads to dysfunction in digestion and absorption but may also increase the body’s susceptibility to pathogens, thereby triggering various diseases such as enteritis [2]. Therefore, maintaining the stability and diversity of the gut microbiota is of great significance to host health [3]. The gut microbiota comprises a highly diverse microbial community dominated by anaerobic bacteria, including members of the phyla Firmicutes, Bacteroidetes, Proteobacteria, Actinobacteria, Fusobacteria, Verrucomicrobia, Cyanobacteria, and Spirochaetes [4,5]. As one of the core phyla, members of Bacteroidetes have particularly complex interactions with the host.

Based on its ability to produce *B. fragilis* toxin (BFT), it can be clearly classified into enterotoxigenic *Bacteroides fragilis* (ETBF) and nontoxigenic *Bacteroides fragilis* (NTBF). BFT is a heat-labile, zinc-dependent metalloprotease of approximately 19–20 kilodaltons (kDa) [6,7]. ETBF exerts its pathogenic effects by secreting BFT, which is its known key virulence factor, playing a significant role in processes such as intestinal inflammation, inflammatory bowel disease (IBD), and colorectal cancer (CRC) [8,9]. In contrast, NTBF does not produce the BFT toxin due to the absence of the *bft* gene [10,11]. This fundamental distinction typically positions NTBF as a safe gut commensal, even demonstrating various beneficial effects for the host [12]. The beneficial effects of NTBF are achieved through multiple pathways: NTBF has immunomodulatory functions, regulating adaptive immune responses via molecular signals such as Polysaccharide A (PSA), balancing pro-inflammatory and anti-inflammatory capabilities [13,14]; it also strengthens the intestinal barrier by enhancing the mucus layer and tight junction structures to consolidate gut barrier integrity [15,16]; it can improve the gut microbiota by employing competition and inhibition strategies to maintain microbial balance and resist pathogen colonization [17,18]; and it may produce selected organic acids and modulate community-level short-chain fatty acid (SCFA) profiles that directly or indirectly affect host health [19,20]. Clarifying the mechanisms underlying the beneficial effects of NTBF is important for assessing its potential benefits and risks and for informing its future development for clinical and veterinary applications [21]. We conducted a structured literature search in PubMed and Web of Science to identify publications on the beneficial effects and potential mechanisms of NTBF. Articles published from January 1992 to June 2026 were included. The search terms included a combination of ‘NTBF’ and ‘*Bacteroides fragilis*’, covering four subject areas: (1) biological characteristics and safety, including genomic characteristics, virulence factors, antimicrobial resistance and toxicological evaluation; (2) core mechanisms, including immune regulation, intestinal barrier protection, and intestinal microbial interactions; (3) metabolites and functional products, including polysaccharide A, outer membrane vesicles and short-chain fatty acids; (4) applications in disease models, including intestinal inflammatory diseases, infectious diseases, colorectal cancer, metabolic disorders, etc. Only English-language journal articles were considered. After duplicate records were removed, titles and abstracts were screened for relevance, followed by full-text assessment. Studies were included if they provided experimental evidence or mechanistic information concerning NTBF biology, safety, functional effects, metabolites, or disease-model applications. Duplicate reports, studies unrelated to NTBF, and studies focused exclusively on ETBF without NTBF-relevant information were excluded. Relevant information was extracted and organized according to the four thematic domains described above. This review summarizes current evidence concerning NTBF as a candidate next-generation probiotic and discusses the challenges of anaerobic cultivation, delivery, host heterogeneity, and long-term safety that must be addressed before clinical translation.

## 2. Biological Characteristics and Safety Basis

Evaluation of the potential beneficial effects of NTBF must be grounded in clearly defined biological characteristics and strain-specific safety evidence. Comprehensive safety evaluation is a prerequisite for any microbial preparation to advance toward clinical or food applications, typically requiring validation from multiple perspectives such as genomic analysis, antibiotic resistance profiling, and preclinical toxicological studies [22,23].

### 2.1. Biological Characteristics

*Bacteroides fragilis* is a Gram-negative obligate anaerobic bacillus and a member of the normal gastrointestinal microbiota, although it can act as an opportunistic pathogen after displacement to extraintestinal sites [24]. In a strain-specific characterization, ZY-312 grew only under anaerobic conditions; formed circular, low-convex, semi-opaque colonies on blood agar; and appeared as Gram-negative rods with rounded ends. The strain was also nonhemolytic [22].

### 2.2. Safety Basis

Genomic analysis is the cornerstone and primary step in assessing the safety of beneficial bacterial strains. Its core purpose is to confirm that the strain does not carry known virulence factors and to evaluate whether its antibiotic resistance genes are located on mobile genetic elements [25]. The most fundamental molecular distinction between NTBF and ETBF lies in the former’s lack of the *bft* gene encoding BFT [26]. This *bft* gene is located within a specific gene cluster known as the *B. fragilis* pathogenicity island (BfPAI), and the complete BfPAI is considered a virulence factor unique to ETBF [11]. In a polymerase chain reaction (PCR)-based molecular epidemiological analysis of *B. fragilis* isolates from blood cultures and other clinical sources, NTBF isolates were classified mainly as pattern II, lacking both the BfPAI and its flanking region, or pattern III, lacking the BfPAI but retaining the flanking region [27]. However, the PCR assay used in that study could not distinguish patterns IV and V from pattern III. For example, a PCR-based epidemiological study of 109 clinical *B. fragilis* isolates collected from hospitals in Rio de Janeiro, Brazil, identified only one ETBF isolate carrying the *bft* gene, whereas 49 isolates (44.9%) were classified as NTBF pattern III. These pattern III isolates lacked the BfPAI but retained conjugative-transposon-associated flanking regions potentially involved in pathogenicity-island acquisition [28]. Therefore, the absence of the BfPAI and the *bft* gene defines the nontoxigenic status of these isolates and removes a major known ETBF virulence determinant. However, it does not establish comprehensive safety or probiotic efficacy, which require separate evaluation for each strain. Recent clinical data further support this distinction. In a Korean cohort of 40 *Bacteroides fragilis* sensu stricto clinical isolates, only 30% carried the *bft* gene, whereas most isolates were *bft*-negative. Among the 34 patients classified as having true infection, intra-abdominal infection was the most common presentation, and 18 of 20 patients with intra-abdominal infection developed *B. fragilis* bacteremia. These findings demonstrate that the absence of *bft* does not exclude extraintestinal pathogenicity, although the study did not directly compare clinical outcomes according to *bft* status [29]. Safety assessment should extend beyond the current absence of the *bft* gene and the intestinal origin of a candidate strain. In a comparative genomic analysis of 813 *Bacteroides fragilis* isolates, including 510 gastrointestinal isolates and 221 isolates from extraintestinal infections, extraintestinal isolates were distributed across multiple phylogenetic lineages, and phylogenetic relatedness was not significantly associated with the anatomical source of isolation. The study also revealed an extensive accessory genome and identified 44 genes statistically associated with the intestinal or extraintestinal source of isolation. These cross-sectional associations do not demonstrate the direct transformation of an individual commensal strain into a pathogen or establish causal roles for the associated genes. Nevertheless, they indicate that intestinal origin, phylogenetic position, and the absence of *bft* are insufficient to define strain safety. Candidate strains should therefore undergo whole-genome assessment of the broader accessory-gene repertoire and mobile genetic elements, together with phenotype-based evaluation of extraintestinal survival and opportunistic pathogenicity [30]. This distinction is also supported by clinical-isolate data showing that the *bft* gene is absent from many *Bacteroides fragilis* isolates recovered from infections, including most isolates in some blood-culture collections. These observations indicate that BFT is not required for extraintestinal infection and that safety evaluation must also consider virulence-associated determinants other than *bft* [31]. Importantly, although *Bacteroides fragilis* is a common intestinal commensal, it can act as an opportunistic pathogen when it translocates beyond the intestinal lumen. The species has been associated with intra-abdominal abscesses, bacteremia, endocarditis, bone and joint infections, and soft-tissue infections. Conditions such as intestinal barrier disruption, dysbiosis, tissue injury, and impaired host immunity may increase the risk of bacterial translocation and extraintestinal infection. Therefore, nonenterotoxigenic status should be regarded only as an initial exclusion criterion rather than sufficient evidence of probiotic safety. A strain-specific safety assessment should include whole-genome screening of known and putative virulence-associated genes, characterization of capsular polysaccharides and abscess-forming potential, antimicrobial-resistance determinants and mobile genetic elements, bacteremia and tissue-translocation risk, potentially adverse metabolic activities, and safety under relevant host conditions, including immunocompromised states [12].

Assessing the antibiotic resistance of NTBF as a beneficial strain requires distinguishing between chromosomally encoded intrinsic resistance and acquired resistance mediated by plasmids or other mobile genetic elements. Mobile genetic elements pose a risk of horizontal gene transfer, potentially spreading resistance to other bacteria (including pathogens) in the gut, thereby raising public health concerns [25,32]. The absence of plasmids alone does not eliminate horizontal-transfer risk, because chromosomal antimicrobial-resistance determinants may still be mobilized through genomic islands, transposons, integrative and conjugative elements, insertion sequences, homologous recombination, or other mobilization machinery. Therefore, transfer-risk assessment should consider not only whether a resistance determinant is located on a chromosome or plasmid, but also its local genomic context, flanking mobile elements, sequence integrity, expression, and experimentally measurable capacity for excision or transfer. The functional importance of the genomic context was illustrated in a clinical collection containing two *cfiA*-positive *Bacteroides fragilis* isolates. The sequence type 157 (ST157) isolate carrying insertion sequence (IS1187) upstream of *cfiA* showed high-level resistance to imipenem and meropenem, whereas another *cfiA*-positive isolate without the upstream insertion sequence remained susceptible. This finding demonstrates that the presence of a resistance gene alone does not reliably predict the phenotype and that candidate strains require analysis of nearby mobile genetic elements together with standardized phenotypic antimicrobial-susceptibility testing [29]. Therefore, whole-genome analysis should evaluate the genomic context and mobility potential of each resistance determinant and should be interpreted together with phenotypic antimicrobial-susceptibility testing [17]. The importance of strain-level phenotypic testing is further supported by a European multicenter surveillance study of 449 *Bacteroides fragilis* isolates recovered from blood cultures. Using a standardized European Committee on Antimicrobial Susceptibility Testing (EUCAST) disc-diffusion method, the study reported resistance rates of 20.9% to clindamycin, 13.4% to meropenem, 11.1% to piperacillin–tazobactam, and 1.8% to metronidazole, with substantial variation among countries. Overall, 35.2% of the isolates were resistant to at least one of the four tested antibiotics, whereas 1.3% met the definition of multidrug resistance. Because the isolates were not classified according to *bft* status and were not candidate probiotic strains, these rates should not be directly attributed to NTBF. Nevertheless, the findings demonstrate that antimicrobial susceptibility in *B. fragilis* cannot be assumed and support standardized phenotypic testing of each candidate strain alongside genomic analysis of resistance determinants and mobile genetic elements [33]. Accordingly, chromosomal location should be regarded as only one component of antimicrobial-resistance assessment. Genotypic predictions should be interpreted together with the database and sequence-matching criteria used, the local genomic context of each determinant, phenotypic antimicrobial-susceptibility results, and evidence distinguishing species-associated intrinsic resistance from acquired resistance [34]. Current evaluations of the NTBF candidate strains ZY-312 and HCK-B3 provide information on the chromosomal location and phenotypic expression of predicted antimicrobial-resistance determinants, but do not establish their complete lack of mobility. Whole-genome analysis of ZY-312 identified 11 putative antimicrobial-resistance genes on the chromosome, with none detected in the reported plasmid sequences. This finding indicates an absence of identified plasmid linkage in the analyzed genome assembly, but it does not exclude mobilization of chromosomal loci through genomic islands, transposons, integrative and conjugative elements, insertion sequences, recombination, or other mechanisms. Moreover, the cited study did not report comprehensive analysis of the flanking genomic context of each resistance determinant or experimental conjugation, transformation, excision, or transfer assays. Phenotypic minimum inhibitory concentration testing showed that ZY-312 was resistant to cefepime, kanamycin, and streptomycin but remained susceptible to several other tested antibiotics, including ceftriaxone, trimethoprim, clarithromycin, chloramphenicol, tetracycline, and levofloxacin [22]. For HCK-B3, genome comparison with the Comprehensive Antibiotic Resistance Database predicted determinants associated with resistance to macrolides, glycopeptides, polymyxin B, chloramphenicol, and tetracycline. Phenotypic testing confirmed resistance to erythromycin, kanamycin, streptomycin, and polymyxin B and susceptibility to penicillin G, cefoxitin, and metronidazole. No plasmids were detected, and the predicted resistance determinants were not located within complete annotated transposons or integrons in the reported analysis [35]. However, the absence of complete annotated mobile elements does not exclude linkage to incomplete, divergent, or unrecognized mobile regions; genomic islands; integrative and conjugative elements; or insertion sequences. Therefore, the available evidence supports chromosomal localization and the absence of identified plasmid linkage for the reported resistance determinants, but the overall horizontal-transfer risk remains incompletely characterized. Long-read genome assembly, detailed analysis of resistance-gene flanking regions, comprehensive mobile-element annotation, and experimental transfer assays under relevant intestinal conditions are required before these loci can be classified as non-mobile or low risk.

The methodological criteria used in the original genomic screens should also be considered. In the ZY-312 study, open reading frames were predicted using Glimmer 3.0 and compared by Basic Local Alignment Search Tool (BLAST) against the Antibiotic Resistance Genes Database (ARDB; originally available at http://ardb.cbcb.umd.edu/). Putative resistance-gene homologs were annotated using a minimum criterion of 40% amino-acid homology. However, the ARDB version and the minimum alignment-coverage threshold were not reported. The original authors also acknowledged that this relatively permissive homology criterion may have contributed to genotype–phenotype discordance and possible false-positive annotation. Therefore, the 11 reported matches are described here as putative resistance-associated homologs rather than as functionally confirmed resistance genes [22].

Phenotypic results were interpreted alongside the genomic predictions. In the ZY-312 study, minimum inhibitory concentration (MIC) testing indicated resistance to cefepime, kanamycin, and streptomycin and susceptibility to ceftriaxone, trimethoprim, clarithromycin, chloramphenicol, tetracycline, and levofloxacin; cefoxitin had a reported MIC of 2 μg/mL. Several predicted homologs, including those associated with trimethoprim, macrolide, phenicol, fluoroquinolone, and tetracycline resistance, were not accompanied by the corresponding resistant phenotype. The study used the breakpoint references available at that time and did not have species-specific interpretive criteria for some tested agents, including vancomycin and polymyxin B. These historical classifications should therefore not be interpreted as a complete contemporary clinical susceptibility assessment [22].

For HCK-B3, the original study reported Comprehensive Antibiotic Resistance Database (CARD)-based genomic prediction together with phenotypic susceptibility testing. However, the published methodological information did not specify the CARD version, sequence-identity threshold, alignment-coverage threshold, or detailed hit-classification criteria sufficiently to permit reproduction of the genomic screen. HCK-B3 was phenotypically resistant to erythromycin, kanamycin, streptomycin, and polymyxin B and susceptible to penicillin G, cefoxitin, and metronidazole [35]. Accordingly, the CARD matches are treated as provisional resistance-associated predictions rather than as confirmed acquired resistance genes.

Intrinsic and acquired resistance could not be conclusively assigned for each predicted determinant in either strain. In this review, intrinsic resistance refers to a species-associated phenotype broadly conserved among *Bacteroides fragilis* strains and not attributable to a recently acquired mobile determinant, whereas acquired resistance refers to strain-specific resistance resulting from horizontally acquired genes or selected resistance mutations. Chromosomal location or sequence similarity to a resistance-gene database alone is insufficient to classify a determinant as intrinsic. Such classification requires comparative analysis across representative strains, evaluation of the genomic neighborhood and mobile elements, and concordant phenotypic or functional evidence. Therefore, the reported determinants in ZY-312 and HCK-B3 are not classified as intrinsic or acquired when the original studies did not provide sufficient evidence for that distinction.

From a clinical perspective, resistance assessment of a candidate NTBF strain should prioritize agents used for the treatment of anaerobic infections, including metronidazole; carbapenems; β-lactam/β-lactamase-inhibitor combinations; cefoxitin; and, where clinically applicable, clindamycin. The reported susceptibility of HCK-B3 to metronidazole and cefoxitin and the low cefoxitin MIC reported for ZY-312 are therefore clinically relevant. However, the available studies did not provide a complete susceptibility panel interpreted using current *Bacteroides-specific* standards, particularly for carbapenems and piperacillin–tazobactam. Consequently, the clinical treatability of these strains in the event of extraintestinal infection remains incompletely characterized and should be reassessed using current standardized anaerobic susceptibility-testing methods and breakpoints.

Furthermore, other studies have revealed potential interactions between NTBF and certain host medications, which is another layer to consider when assessing its application safety: A short-term metagenomic study showed that fecal *B. fragilis* abundance decreased in newly diagnosed patients with type 2 diabetes after 3 days of metformin treatment; however, the affected strains were not characterized as NTBF or ETBF [36]. Aryl coumarin derivatives have been shown to inhibit the activity of *B. fragilis* d-lactate dehydrogenase (Bfd-LDH), an enzyme that plays a key role in the bacterial anaerobic glycolysis pathway [37]. Aspirin treatment was associated with reduced fecal *B. fragilis* abundance in mouse models of colorectal carcinogenesis, although the affected strains were not characterized as NTBF or ETBF and were not shown to mediate aspirin degradation [38]. In another study, the bovine-derived NTBF strain BF-1153, isolated from normal calf feces, exhibited resistance to lincomycin, erythromycin, azithromycin, tetracycline, and doxycycline in an anaerobic disc-diffusion susceptibility assay, broadly consistent with resistance genes predicted from ARDB and CARD annotations [39]; these findings suggest that when developing NTBF into probiotic products in the future, potential interactions with commonly used host medications need to be fully evaluated.

Preclinical toxicological studies aim to systematically assess a strain’s acute toxicity, subchronic toxicity, genotoxicity, teratogenicity, and potential effects on specific organ functions through a series of standardized in vitro and in vivo experiments. This is a crucial step in ensuring the application safety of any candidate probiotic strain [23]. In vitro studies often serve as preliminary screening, focusing on assessing the strain’s basic biocompatibility and its direct effects on the intestinal barrier. Additionally, assessing the strain’s survival ability in the digestive tract through tolerance tests to gastric acid and bile salts, as well as adhesion ability tests to intestinal epithelial cells, are common in vitro evaluation items, helping to predict its potential for effective colonization in the gut [22]. In a C57BL/6 mouse model with antibiotic-induced gut microbiota disruption, the live ZY-312-only group received live *B. fragilis* ZY-312 by oral gavage at 1 × 10^8^ colony-forming units (CFU)/day from day −9 to day 2 without *C. difficile* challenge. No deaths occurred, and histopathological and immunohistochemical assessments of the cecum and colon did not indicate overt intestinal tissue injury. However, three of six mice developed transient mild diarrhea on day 2 and recovered by day 3. These findings support the short-term tolerability of live ZY-312 under the specific experimental conditions used in this study [40]. In a 28-day study, female C57BL/6J mice were orally administered a live sheep-derived *B. fragilis* isolate at either 1 × 10^7^ or 2 × 10^8^ CFU per mouse per day. Mice receiving the higher dose, designated the BF109 group, had a significantly greater final body weight than phosphate-buffered saline (PBS)-treated controls, whereas the same effect was not reported for the lower-dose BF108 group. No overt pathological alterations were observed in the examined intestinal tissues. The authors interpreted the increased body weight as a potential improvement in growth performance; however, energy metabolism was not directly assessed, and the enterotoxigenic status of the tested isolate was not established in this study [41]. Specialized reproductive and developmental toxicity studies may also strengthen the preclinical safety assessment of probiotic candidates. For example, a concentrated powder preparation of the commercially used probiotic strain *Clostridium butyricum* MIYAIRI 588^®^ was evaluated in pregnant Institute of Cancer Research (ICR) mice and showed no dose-related adverse effects on reproduction or offspring development under the tested conditions [42]. Assessment of bacterial translocation and systemic dissemination is particularly critical for evaluating the safety of obligate anaerobes, requiring confirmation of whether they might escape the gut and enter the bloodstream or visceral organs under specific conditions. Xu et al. used metabolic oligosaccharide engineering and bioorthogonal click chemistry to fluorescently label live NTBF strain ZY-312 and track its distribution after oral gavage in C57BL/6 mice. In vivo imaging showed that the fluorescent signal moved from the stomach and small intestine to the cecum and colon and remained detectable, particularly in the cecum, for up to 48 h. Immunofluorescence analysis further detected labeled ZY-312 in the stomach, ileum, cecum, and colon at 24 and 48 h, whereas no obvious fluorescent signal was detected in the spleen or kidneys at the examined time points [43]. The absence of an obvious fluorescent signal in the spleen and kidneys supports a lack of detectable dissemination to these organs at the examined time points. However, the study did not exclude low-level translocation, dissemination to unexamined tissues, or distribution beyond the 48-h observation period.

The assessment of immunogenicity and safety under immunodeficient conditions is equally indispensable, as it relates to the applicability of the strain in populations with normal or compromised immune function. In the study of the HCK-B3 strain, experiments utilized both healthy male C57BL/6 mice and cyclophosphamide-induced immunodeficient mouse models. Researchers evaluated five aspects: the general clinical condition of the animals, hematological parameters, serum biochemical indicators, histopathological examination of major organs, and systemic inflammatory cytokine levels. Across all evaluation metrics, oral administration of HCK-B3 did not induce any significant adverse reactions or signs of toxicity in either healthy or immunodeficient mice [35]. A separate study investigated the functional role of systemic immunoglobulin A (IgA) induced by intestinal colonization with the live nontoxigenic *B. fragilis* strain NCTC 9343. Wild-type C57BL/6J and IgA-deficient mice received repeated oral gavage with live NCTC 9343 for 10 weeks and were subsequently challenged intraperitoneally with live *B. fragilis* mixed with heat-inactivated cecal slurry in a murine peritoneal abscess model. NCTC 9343-pretreated wild-type mice, which developed high levels of strain-specific serum IgA, exhibited significantly fewer peritoneal abscesses than untreated wild-type controls or similarly pretreated IgA-deficient mice. These findings support an IgA-dependent protective effect against abscess formation under this specific experimental condition [44]. These findings support an association between sustained intestinal colonization during repeated live NCTC 9343 administration and the induction of a strain-specific systemic IgA response that reduced abscess formation in this murine challenge model. However, the repeated-dosing design should be distinguished from long-term colonization after treatment withdrawal. The study supports a colonization-dependent immune response under its specific experimental conditions but should not be interpreted as demonstrating permanent colonization or as establishing that colonization is required for all NTBF-associated effects [44].

Beyond conventional toxicological and translocation endpoints, potentially adverse metabolic effects should also be evaluated under disease-relevant dietary and host conditions. In male specific-pathogen-free C57BL/6J mice fed a high-fat diet for 16 weeks, oral administration of live *Bacteroides fragilis* ATCC 25285 at 2 × 10^8^ CFU every other day during the final 8 weeks further increased body weight and adipose tissue mass, worsened fasting glucose and serum lipid profiles, elevated serum alanine aminotransferase and lipopolysaccharide levels, and aggravated hepatic steatosis and hepatocellular ballooning compared with high-fat-diet controls. These findings indicate that the metabolic effects of a candidate strain may depend on the host dietary and metabolic context and should therefore be included in strain-specific safety assessment [45].

In summary, through evaluations in genomics, antibiotic resistance, and preclinical toxicology, current research provides data supporting the safety of representative NTBF strains such as ZY-312 and HCK-B3. These efforts lay a theoretical foundation for their subsequent advancement to clinical trial stages and the eventual realization of their potential applications as probiotics or live biotherapeutic products.

## 3. Strain-Level Heterogeneity of the Beneficial Effects of NTBF

Although the absence of the *bft* gene and the corresponding lack of BFT production define the nontoxigenic status of *Bacteroides fragilis*, this classification alone does not establish a uniform functional phenotype. Direct comparisons performed under the same experimental conditions demonstrate strain-dependent differences in functional capacity. In the study of SNBF-1, 38 non-toxigenic *B. fragilis* isolates were initially screened, after which seven isolates, including SNBF-1 and SY-X-3, were selected for further characterization. SNBF-1 exhibited an in vitro cholesterol removal rate of 55.38 ± 2.26%, compared with 22.08 ± 1.04% for SY-X-3, corresponding to a calculated ratio of approximately 2.51. Bile salt hydrolase activity was 410.04 ± 12.29 U/L for SNBF-1 and 316.79 ± 9.11 U/L for SY-X-3, corresponding to a calculated ratio of approximately 1.29 [21]. These results provide direct quantitative evidence that metabolic capacity is not uniform among NTBF strains. However, these in vitro measurements demonstrate only strain-dependent differences in bile salt hydrolase activity. The study did not determine substrate specificity, characterize the resulting bile-acid profile, or evaluate the corresponding effects in vivo. Therefore, higher bile salt hydrolase activity should not be interpreted as evidence of greater probiotic benefit.

Strain-dependent differences were also observed in host responses. In a lipopolysaccharide (LPS)-treated mouse model, NCTC 9343 (ATCC 25285) increased phosphorylated inhibitor of κB (IκB) by approximately 50% relative to the LPS control, whereas HCK-B3 did not further increase phosphorylated IκB; the difference between the two strains was statistically significant (*p* < 0.05). In the same study, HCK-B3 reduced serum tumor necrosis factor alpha (TNF-α) (*p* < 0.05), increased interleukin-10 (IL-10) (*p* < 0.01), and restored the proportion of splenic regulatory T (Treg) cells reduced by LPS (*p* < 0.01) [46]. These direct comparisons document different immunological and signaling responses among *B. fragilis* strains under the same experimental conditions.

In the studies summarized here, direct quantitative comparisons of PSA production or structure and type VI secretion system (T6SS) effector repertoires across representative NTBF strains were not reported. For ZY-312, the cited studies evaluated a purified strain-specific zwitterionic capsular polysaccharide preparation termed TP2, with an average relative molecular mass of approximately 70 kDa. Although the source articles sometimes referred to TP2 as PSA, direct biochemical equivalence with canonical NCTC 9343 PSA was not established. TP2 was therefore evaluated and is described here as a distinct ZY-312-derived polysaccharide preparation [47]. For HCK-B3, four PSA-biosynthesis-associated genes, *upaY*, *upaZ*, *wcfR*, and *wcfS*, showed 98–100% sequence similarity to their counterparts in NCTC 9343 (ATCC 25285); however, PSA production, structure, and biological activity were not experimentally evaluated. Genome analysis of HCK-B3 identified the putative T6SS-associated genes *clpV* and *clpB*, whereas the core components VgrG and Hcp were absent; no functional T6SS assay was reported [35]. These findings show that the currently available PSA and T6SS evidence differs in type and completeness among the representative strains and does not establish equivalent PSA or T6SS activity across NTBF strains.

Table 1 therefore distinguishes experimentally demonstrated functions from genome-predicted and uncharacterized features. Representative strains, including ZY-312, NCTC 9343 (ATCC 25285), HCK-B3, SNBF-1, and BF-1153, are compared according to their source, *bft* status, PSA or capsular-polysaccharide evidence, T6SS or bacteriocin evidence, quantitative and functional findings, and experimental models. This comparison emphasizes that nontoxigenic status alone does not demonstrate a shared probiotic phenotype and that the functional properties of each strain must be evaluated using strain-specific experiments.

As summarized in Table 1, the available functional and preclinical evidence is unevenly distributed across NTBF strains and experimental preparations. Among the strains included, ZY-312 has been evaluated in several intestinal disease models. Live ZY-312 promoted group 3 innate lymphoid cell (ILC3)-derived interleukin-22 (IL-22) production and IL-22-dependent signal transducer and activator of transcription 3 (STAT3) phosphorylation in dextran sulfate sodium (DSS)-induced colitis and exerted preventive effects in a *Cronobacter sakazakii*-induced neonatal rat model of necrotizing enterocolitis. In contrast, TP2 is a purified, ZY-312-derived zwitterionic capsular polysaccharide preparation that was evaluated separately in TNBS-induced colitis. Although the source studies sometimes termed TP2 PSA, its biochemical equivalence with canonical NCTC 9343 PSA was not established by direct comparative analysis. Therefore, the effects of TP2 should not be attributed either to live ZY-312 or to canonical NCTC 9343 PSA. NCTC 9343 (ATCC 25285) has been investigated in TNF-α-induced inflammatory injury in human colonic epithelial cells and in DSS-induced acute colitis in mice. The reported effects include attenuation of nuclear factor kappa B (NF-κB) activation and epithelial injury, regulation of inflammatory cytokines, increased expression of tight-junction proteins, and alterations in fecal SCFA profiles. HCK-B3 has complementary genomic, functional, and safety evidence. Whole-genome analysis did not detect *bft* and identified conserved PSA-biosynthesis-associated genes, although PSA production and activity were not experimentally confirmed. Its putative T6SS repertoire was incomplete, with *clpV* and *clpB* detected but VgrG and Hcp absent. In a five-day pilot study, mice received live HCK-B3 at 1 × 10^9^ CFU/day, either alone or together with intraperitoneal LPS at 0.1 mg/kg. HCK-B3 reduced serum TNF-α, increased IL-10, restored splenic regulatory T cells, and counteracted selected LPS-associated microbiota alterations. However, it also significantly increased serum fluorescein isothiocyanate–dextran (FITC–dextran) concentrations in both healthy and LPS-treated mice. The published figure, which did not provide exact tabulated values, showed Dextran-FITC levels of approximately 220% of the untreated blank level in HCK-B3-treated healthy mice, compared with approximately 120% in vehicle-treated controls, and approximately 240% in HCK-B3-plus-LPS mice, compared with approximately 170% in LPS-treated controls. Colonic histology showed no obvious mucosal disruption, and serum LPS concentrations were comparable among the treatment groups. Nevertheless, permeability was assessed only once at the end of the five-day intervention, using a single HCK-B3 dose in one pilot study. Therefore, the duration, reversibility, dose dependence, biological consequences, and independent reproducibility of this finding remain unknown. Transient epithelial stress, inflammatory activation, a dose-related effect, or toxicity specific to the LPS model cannot be excluded. The increased permeability should therefore be regarded as a potential safety signal rather than evidence of beneficial immune priming [46]. A separate safety study found no evident adverse effects following oral administration to healthy or cyclophosphamide-immunosuppressed mice. Evidence for SNBF-1 is predominantly derived from quantitative in vitro assays, including cholesterol removal, bile salt hydrolase activity, modulation of lipid and glucose metabolism in HepG2 cells, and antioxidant activity. BF-1153 was isolated from normal calf feces, but its functional validation was performed using cell-line assays and a BRV-induced diarrhea model in specific-pathogen-free (SPF) Kunming mice. Therefore, the existing study supports its preliminary veterinary probiotic potential but does not demonstrate efficacy or intestinal microbiota regulation in cattle. Collectively, these findings show that nontoxigenic status cannot be used as a surrogate for a shared probiotic phenotype. The functional profile of each candidate NTBF strain must instead be defined by strain-specific molecular characteristics, quantitative functional measurements, the experimental preparation used, and performance in clearly specified disease models.

## 4. Mechanistic Evidence and Proposed Pathways

**Figure 1:**
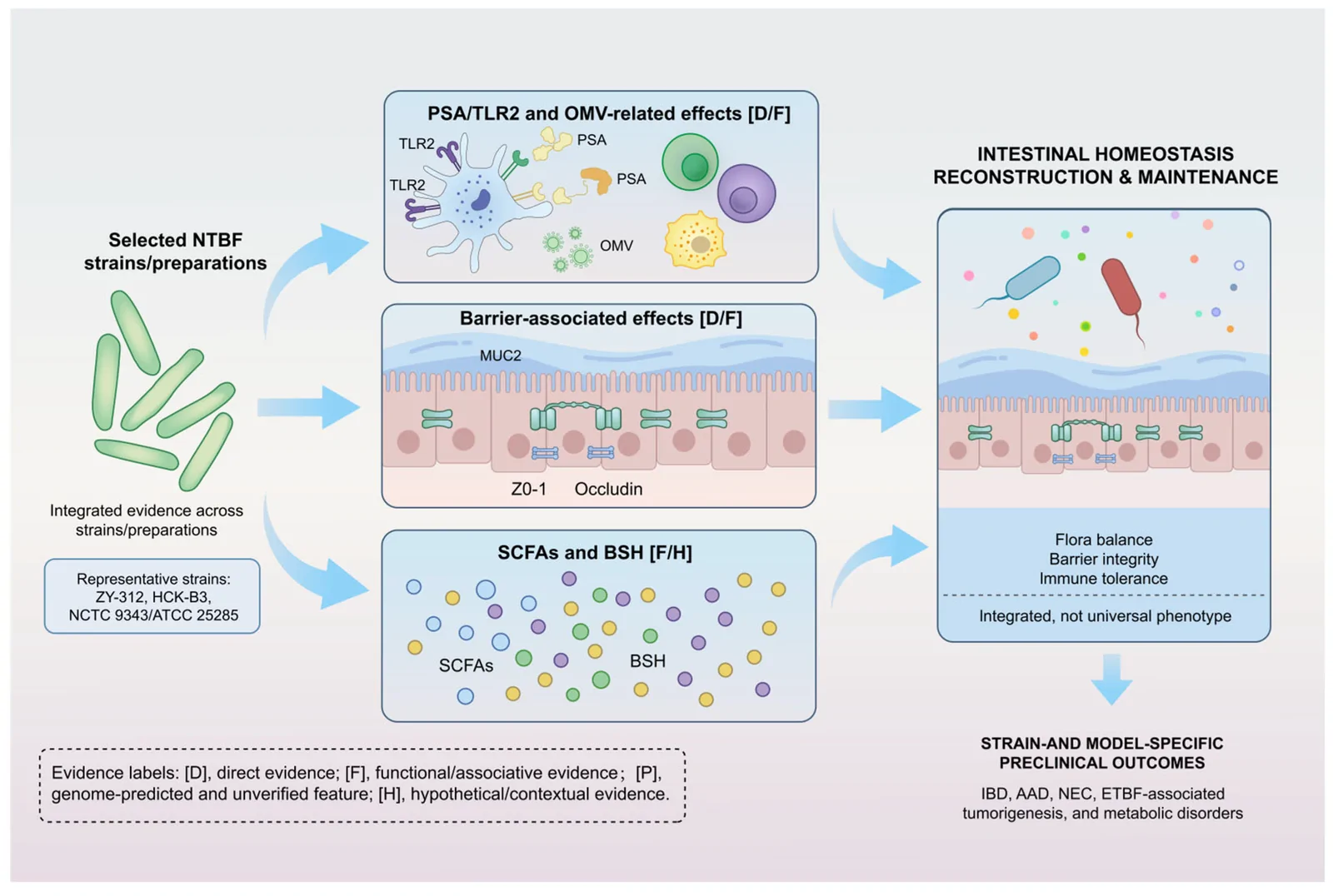
Integrated overview of evidence reported for selected NTBF strains and strain-derived preparations. The illustrated immune, barrier, metabolic, and disease-related effects derive from different strains, preparations, and experimental models and do not represent a universal NTBF phenotype [16,21,47,49,51,52,53,54,55,56]. Abbreviations: NTBF, nontoxigenic *Bacteroides fragilis*; PSA, polysaccharide A; TLR2, Toll-like receptor 2; OMV, outer membrane vesicle; MUC2, mucin 2; ZO-1, zonula occludens-1; SCFAs, short-chain fatty acids; BSH, bile salt hydrolase; IBD, inflammatory bowel disease; AAD, antibiotic-associated diarrhea; NEC, necrotizing enterocolitis; ETBF, enterotoxigenic *Bacteroides fragilis*; NCTC, National Collection of Type Cultures; ATCC, American Type Culture Collection. Evidence labels: [D], direct causal evidence; [F], functional or associative evidence; [P], genome-predicted and unverified feature; [H], hypothetical or contextual pathway. The blue, yellow, green, and purple circles schematically represent unspecified surrounding cells in the local tissue microenvironment; the different colors do not indicate distinct cell types or functions.

Experimental evidence reviewed in this section identifies three recurrent mechanistic domains among selected NTBF strains and their derived preparations: immune regulation, effects on intestinal barrier-associated features, and microbial competition or community modulation. The molecular pathways involved differ according to the bacterial strain, experimental preparation, administered dose, host, and disease model and are not uniformly shared across NTBF. To distinguish established mechanisms from associative or extrapolated observations, the evidence reviewed in this section was classified into three levels according to causal strength. Level I, direct mechanistic evidence, requires a defined strain or purified molecule together with a causal intervention or comparison, such as an isogenic knockout or complemented strain, a receptor-deficient or conditional-knockout animal, pathway inhibition, gene knockdown or knockout, a rescue experiment, or a purified-molecule study that directly validates the relevant molecular target or pathway. Level II, preparation-specific functional evidence, includes reproducible changes observed after administration of live bacteria, outer membrane vesicles, culture supernatants, or purified fractions, such as changes in cytokine levels, signaling-protein phosphorylation, gene or protein expression, histological injury, epithelial permeability, or disease severity, when the proposed causal pathway has not been directly tested. Level III, indirect or contextual evidence, includes microbiota or metabolite correlations, community-level changes, general intestinal physiology, and findings extrapolated from unrelated probiotic species. Only Level I evidence is described as establishing a mechanism, whereas Level II and Level III findings are described as preparation-specific functional effects, associations, or proposed pathways. The integrated overview of these strain- and preparation-specific mechanistic domains is presented in Figure 1.

### 4.1. Immune-Regulating

In this review, the term “canonical PSA” refers specifically to the structurally characterized capsular polysaccharide A of *Bacteroides fragilis* NCTC 9343 (ATCC 25285). Canonical PSA is a zwitterionic polysaccharide composed of branched tetrasaccharide repeating units containing 2-acetamido-4-amino-2,4,6-trideoxygalactose and a 4,6-O-pyruvylated galactose residue. The free amino group and the pyruvate-derived carboxyl group confer its characteristic oppositely charged structure [57,58]. Chemical modification of these charged groups markedly reduces its T-cell-stimulatory and protective activities, demonstrating the functional importance of the zwitterionic motif [58]. Native NCTC 9343-derived PSA had an average molecular mass of approximately 129 kDa, and fragments of 17.1 kDa retained cluster of differentiation 4 (CD4)^+^ T-cell-stimulatory and abscess-protective activity, whereas 5.0-kDa fragments were substantially less stimulatory and failed to confer protection [59]. Following uptake by antigen-presenting cells, PSA is oxidatively depolymerized into lower-molecular-weight fragments, loaded onto major histocompatibility complex class II (MHCII), and presented to CD4^+^ T cells [60]. More recent evidence indicates that PSA-mediated immunomodulation requires coordinated Toll-like receptor 1/2 (TLR1/TLR2) and dendritic cell-associated C-type lectin-1 (Dectin-1) signaling and a covalently attached lipid moiety that is critical for antigen-presenting-cell activation and anti-inflammatory responses [51]. These detailed structure–function relationships were established primarily using NCTC 9343-derived PSA or a PSA-overexpressing *B. fragilis* preparation and should not be assumed to apply identically to PSA-like polysaccharides from all NTBF strains.

TP2 is treated in this review as a distinct strain-specific polysaccharide preparation. The source studies describe TP2 as a purified zwitterionic capsular polysaccharide isolated from *Bacteroides fragilis* ZY-312, with an average relative molecular mass of approximately 70 kDa. The reported preparation contained approximately 98% total sugar, 1% protein, and 0.5% nucleic acid. Its repeating unit was reported to contain 2,4-dideoxy-4-amino-D-N-acetylfucose, D-N-acetylgalactosamine, D-galactopyranose, and D-galactofuranose, with a 4,6-pyruvate group attached to the galactopyranose. The source article stated that TP2 was isolated and purified according to a previously described procedure but did not provide a complete step-by-step purification protocol in the cited experiment. Although some source articles refer to TP2 as PSA, no direct side-by-side structural, immunochemical, or functional equivalence analysis between TP2 and canonical NCTC 9343 PSA was reported. Accordingly, TP2 is referred to throughout this review as “ZY-312-derived zwitterionic capsular polysaccharide TP2” and is not used interchangeably with canonical PSA. Findings obtained using purified TP2 are attributed specifically to the TP2 preparation and should not be attributed to live ZY-312 or canonical NCTC 9343 PSA [47].

The immune-regulatory effects reviewed in this section were demonstrated using specific NTBF strains or strain-derived preparations and should not be interpreted as properties uniformly shared by all NTBF strains. PSA has been demonstrated by research to indirectly induce the differentiation and function of regulatory T cells (Tregs), thereby playing a central role in maintaining immune balance and alleviating intestinal as well as systemic inflammation [13,14]. In experimental systems using purified PSA, MHCII-dependent activation of PSA-responsive CD4^+^ T cells may indirectly support Treg-mediated immunosuppression through interactions with tissue-resident Tregs. PSA is a capsular polysaccharide of *B. fragilis* with context-dependent, T-cell-mediated pro- and anti-inflammatory properties [61]. One documented pathway of PSA-mediated immune regulation involves TLR2-dependent activation of plasmacytoid dendritic cells (pDCs). In the murine experimental system summarized by Kayama and Takeda, PSA-treated pDCs, rather than conventional dendritic cells, promoted IL-10-producing Treg responses through cognate interactions with CD4^+^ T cells that required the expression of MHCII, cluster of differentiation 86 (CD86), and inducible T-cell costimulator ligand (ICOS-L) on pDCs, thereby contributing to protection against TNBS-induced intestinal inflammation. However, this pathway was characterized primarily using bone marrow-derived pDCs, and whether gut-resident pDCs mediate an equivalent response under physiological conditions remains to be determined [62]. Consistent with these murine findings, purified PSA promoted dendritic-cell-dependent induction of cluster of differentiation 39 (CD39)^+^ forkhead box P3 (Foxp3)^+^ T cells from human naïve CD4^+^ T cells in vitro, increased IL-10 secretion, and enhanced suppressive Treg function, although the magnitude of the response varied among donors [63].

The dynamic balance between T helper 17 cells (Th17) and regulatory T cells (Treg) is conducive to maintaining immune homeostasis. Th17 cells are characterized by the production of the pro-inflammatory cytokine interleukin-17 (IL-17), and dysregulated Th17/IL-17 responses are associated with autoimmune disease and chronic inflammation [64]. In contrast, Treg cells actively inhibit effector T cell responses by secreting cytokines such as IL-10 and transforming growth factor-β (TGF-β) and are central to maintaining immune tolerance and preventing autoimmunity [65]. In necrotizing enterocolitis, intestinal immune dysregulation is characterized by an increased pro-inflammatory Th17-cell response and a reduced proportion of Treg cells, and this Th17/Treg imbalance contributes to the exaggerated inflammatory response in the intestine [66]. In the experimental systems summarized by Shen et al., *B. fragilis*-derived PSA modulated the intestinal Th17/Treg balance by increasing intestinal Treg numbers, promoting their local expansion, and enhancing their suppressive phenotype, while reducing IL-17 production and mucosal Th17 responses. However, these findings were derived from studies involving PSA-producing *B. fragilis*, PSA-deficient bacterial counterparts, or orally administered purified PSA and should not be interpreted as a mechanism uniformly shared by all NTBF strains [67]. Beyond its intestinal effects, orally administered purified *B. fragilis* PSA has been shown to modulate distal inflammation in the murine experimental autoimmune encephalomyelitis model. In this setting, PSA promoted tissue-specific CD39^+^ regulatory CD4^+^ T-cell responses, increased IL-10 production, reduced IL-17-associated inflammation, and attenuated central nervous system inflammation and demyelination through TLR2- and CD39-dependent mechanisms [68]. NF-κB is a major transcriptional regulator downstream of inflammatory signals such as tumor necrosis factor (TNF) and induces the expression of cytokines, chemokines, receptors, adhesion molecules, and other inflammation-associated genes. Dysregulated TNF–NF-κB/mitogen-activated protein kinase (MAPK) signaling has been implicated in inflammatory disorders, including IBD, although TNF-driven inflammation may also involve receptor-interacting serine/threonine-protein kinase 1 (RIPK1)-dependent cell-death pathways [69]. Mechanistically, PSA-mediated regulation of NF-κB appears to involve downstream signaling reprogramming rather than simple competitive receptor blockade. In antigen-presenting-cell-based systems, PSA engages TLR2/TLR1 in cooperation with Dectin-1 and activates the phosphoinositide 3-kinase (PI3K)/protein kinase B (Akt) pathway. Akt-mediated phosphorylation maintains glycogen synthase kinase 3 beta (GSK3β) in an inactive state, thereby limiting NF-κB/cAMP response element-binding protein (CREB)-binding protein (CBP)-dependent pro-inflammatory transcription and favoring CREB/CBP-dependent anti-inflammatory gene expression and IL-10 production. Because PSA can still induce inhibitor of κB alpha (IκBα) degradation, this mechanism does not represent complete inhibition of proximal TLR–NF-κB activation. Moreover, direct competition with LPS for receptor binding and the induction of negative regulators such as tumor necrosis factor alpha-induced protein 3 (A20) or interleukin-1 receptor-associated kinase M (IRAK-M) have not been demonstrated [51]. In the LPS-stimulated human macrophage-like THP-1 cell model, activation of the Toll-like receptor 4 (TLR4)-associated myeloid differentiation primary response 88 (MyD88)–IRAK–tumor necrosis factor receptor-associated factor 6 (TRAF6)–transforming growth factor-beta-activated kinase 1 (TAK1)–IκB kinase (IKK) signaling cascade promotes IκB degradation and the phosphorylation and nuclear translocation of NF-κB p65. Activated NF-κB subsequently induces the transcription of inflammation-associated genes, including interleukin-1 beta (IL-1β), TNF-α, interleukin-6 (IL-6), C-C motif chemokine ligand 2 (CCL2), and cyclooxygenase-2 (COX-2) [70]. In a separate LPS-induced hepatic inflammation model, purified PSA isolated from *B. fragilis* NCTC 9343 reduced the expression of TLR4/MyD88/TRAF6/NF-κB pathway-associated molecules and decreased IL-1β expression. TLR4 inhibition and knockdown experiments suggested that this effect was associated with the TLR4 pathway; however, the proximal mechanism, including possible receptor competition or induction of intracellular negative-feedback regulators, was not determined [71]. In a TNF-α-stimulated human colonic epithelial-cell model, pretreatment with live *B. fragilis* ATCC 25285 attenuated NF-κB activation, as indicated by reduced phosphorylation of NF-κB p65 and IκBα, and was accompanied by reduced IL-6 and IL-1β responses and increased IL-10 production. Although the study also measured TLR2, TLR4, and MyD88, the involvement of TLR signaling in the TNF-α-induced response remained unclear. A filtered cell-free culture supernatant from ATCC 25285 produced partly similar effects on IL-6, IL-1β, and IL-10 but increased interleukin-8 (IL-8), suggesting that the effects of the live bacterium and its extracellular products may not be identical. Moreover, the study did not determine whether the observed anti-inflammatory effects were PSA-dependent [49]. In an independent study using NCTC 9343, wild-type *B. fragilis* protected against *Helicobacter hepaticus*-induced colitis, whereas a PSA-deficient mutant did not provide comparable protection. Purified PSA was also sufficient to alleviate *H. hepaticus*- and TNBS-induced experimental colitis [72]. In contrast, both wild-type NCTC 9343 and its isogenic ΔPSA mutant provided similar prophylactic protection against ETBF-induced chronic colitis and tumor progression when administered before ETBF challenge [73]. Thus, the contribution of PSA is model- and context-dependent, and the anti-inflammatory effects of intact *B. fragilis* cannot be attributed exclusively to PSA.

Recent human-cell evidence further indicates that the immunological effects of NTBF-derived outer membrane vesicles are preparation specific and are not uniformly anti-inflammatory. In a human gastrointestinal multicellular model comprising Caco-2, HT29-MTX, and Raji B cells, outer membrane vesicles derived from nontoxigenic *Bacteroides fragilis* NCTC 9343 and enterotoxigenic *B. fragilis* 86-5443-2-2 were evaluated at 10 and 100 μg/mL after 2-h exposure. No significant concentration-dependent difference was detected between the two tested doses, and the reported analyses therefore focused mainly on the 10 μg/mL condition. NCTC 9343-derived outer membrane vesicles produced significant changes in several innate-cell-recruitment chemokines and growth-factor-associated proteins relative to the vehicle control and induced a broader cytokine response than the ETBF-derived preparation, whereas statistically significant changes were not consistently observed across the interleukin group. However, the measured protein concentrations were low, the experiment was limited to an in vitro multicellular system, and no receptor-specific intervention, functional epithelial-barrier assay, or in vivo validation was performed. These findings therefore represent preparation-specific immune activation rather than evidence of a uniformly beneficial or anti-inflammatory NTBF mechanism [74].

**Figure 2:**
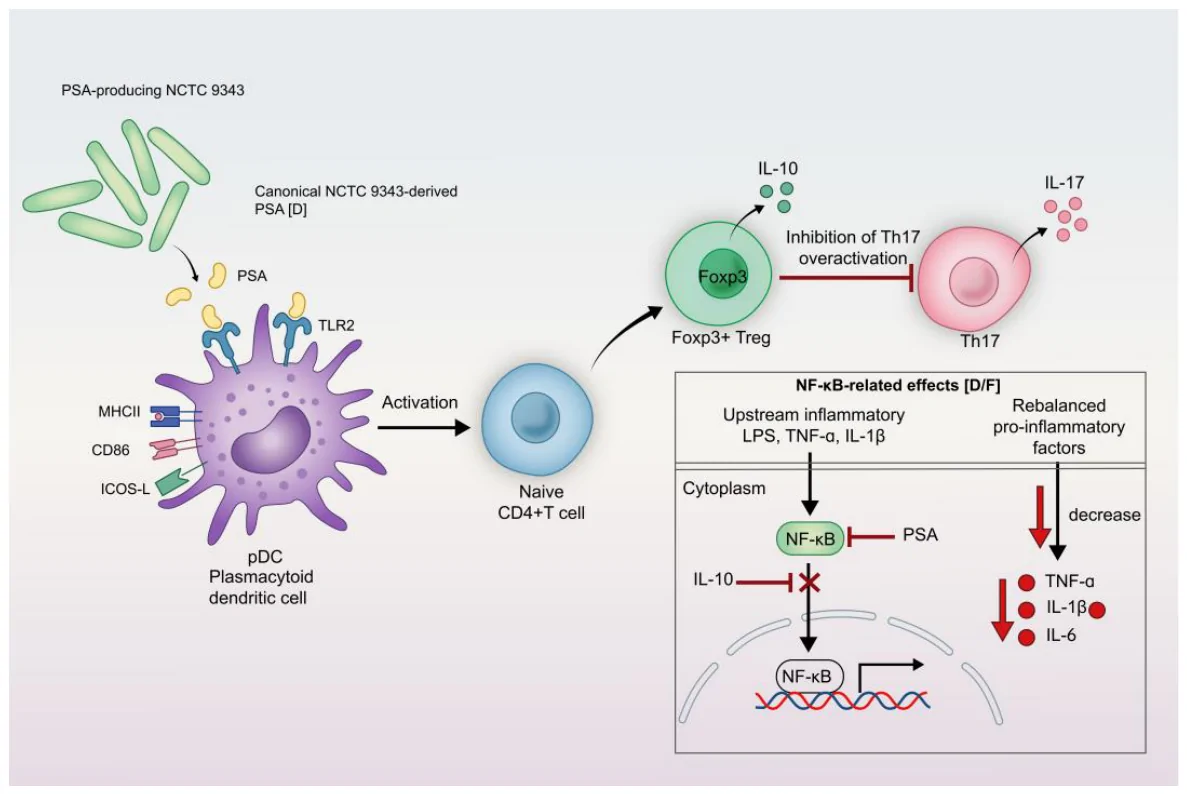
Immune-regulatory effects reported for canonical NCTC 9343-derived PSA and defined NCTC 9343/ATCC 25285 preparations [49,51,62,63,68,71,72]. These pathways have not been demonstrated uniformly across NTBF strains. Abbreviations: PSA, polysaccharide A; NCTC, National Collection of Type Cultures; ATCC, American Type Culture Collection; TLR2, Toll-like receptor 2; MHCII, major histocompatibility complex class II; CD4, cluster of differentiation 4; CD86, cluster of differentiation 86; ICOS-L, inducible T-cell costimulator ligand; pDC, plasmacytoid dendritic cell; Foxp3, forkhead box P3; Treg, regulatory T cell; Th17, T helper 17 cell; IL, interleukin; NF-κB, nuclear factor kappa B; LPS, lipopolysaccharide; TNF-α, tumor necrosis factor alpha. Evidence labels: [D], direct causal evidence; [F], functional or associative evidence. Arrows indicate activation or promotion, T-shaped blunt-ended lines indicate inhibition or negative regulation, and the “×” symbol indicates blockade of the corresponding pathway, interaction, or translocation.

The reported immunomodulatory effects of specific NTBF strains and strain-derived preparations should be considered within the broader context of intestinal homeostasis. Under physiological conditions, coordinated interactions among the gut microbiota, intestinal epithelial barrier, and mucosal immune system maintain immune tolerance and barrier integrity. A similar immune–barrier pattern has been reported for other microbiota-targeted interventions. In a DSS-induced chronic colitis model, Schisandra chinensis bee pollen extract (SCPE) and fecal microbiota transplantation (FMT) from SCPE-treated donor mice restored Treg/Th17 balance; reduced IL-1β, IL-6, and IL-17 levels; increased IL-10; and enhanced ZO-1 and occludin expression [75]. However, this study did not investigate NTBF and therefore cannot be used as direct evidence for NTBF-mediated immune regulation. Collectively, PSA is an important immunomodulatory effector in selected PSA-characterized *B. fragilis* strains. In defined experimental systems, purified PSA or PSA-producing bacteria can promote regulatory CD4^+^ T-cell responses, enhance IL-10 production, reduce IL-17-associated inflammation, and modulate NF-κB-related signaling. However, these effects are strain-, preparation-, and model-dependent, and the effects of intact bacteria may also involve PSA-independent components or metabolites. Therefore, PSA should not be regarded as a universal or exclusive mechanism of immune regulation across all NTBF strains. The immune-regulatory effects reported for defined NTBF strains and strain-derived preparations are summarized in Figure 2.

### 4.2. Protecting the Intestinal Barrier

Barrier-associated effects have been reported for defined live NTBF strains and strain-derived preparations rather than for NTBF as a uniform category. In specific experimental models, live ZY-312, live ATCC 25285, ZY-312-derived TP2, and ATCC 25285-derived outer membrane vesicles were associated with changes in goblet-cell numbers, mucin 2 (MUC2) expression, tight-junction-associated proteins, epithelial proliferation, or intestinal permeability. Direct causal evidence is strongest for the epithelial STAT3 dependence of live ZY-312 in the radiation-induced intestinal-injury model. In other studies, changes in goblet-cell numbers, mucus-associated markers, or tight-junction proteins should be interpreted as preparation- and model-specific barrier-associated effects unless the corresponding causal pathway was directly tested. The intestinal mucus layer is a gel-like network formed by mucin MUC2 secreted by goblet cells, and it serves as the first physical and chemical line of defense for the host against pathogens and harmful substances [76]. This barrier not only prevents direct contact between microorganisms and the epithelium through physical separation, but also actively inhibits pathogen colonization through substances it contains, such as antimicrobial peptides [77]. Evidence for mucus-barrier effects differs in causal strength among strains and experimental preparations. The ZY-312 IL-22–STAT3 study provides direct mechanistic evidence because interruption of epithelial STAT3 signaling attenuated the barrier-associated response. In contrast, changes in goblet-cell numbers, MUC2 expression, apoptosis-associated proteins, or tight-junction-associated proteins following treatment with TP2, outer membrane vesicles, or whole-bacterium preparations represent preparation-specific functional evidence unless the corresponding causal pathway was directly interrupted or rescued. In a TNBS-induced colitis model in male Wistar rats, treatment with the purified ZY-312-derived zwitterionic capsular polysaccharide TP2 alleviated the loss of colonic goblet cells, as demonstrated by periodic acid–Schiff (PAS) staining and quantitative analysis. In mechanistic analyses using the 2 mg/kg TP2 group, treatment increased the B-cell lymphoma 2 (Bcl-2)/Bcl-2-associated X protein (Bax) ratio and reduced cleaved caspase-3 expression in colonic tissue. These findings indicate that ZY-312-derived TP2 was associated with reduced colonic apoptosis and restoration of barrier-associated features under the conditions of this model [47]. In a DSS-induced colitis mouse model, treatment with live nontoxigenic *B. fragilis* ATCC 25285 alleviated colonic goblet-cell depletion. Pharmacological enhancement of outer membrane vesicle (OMV) secretion by the same strain further strengthened this effect, whereas inhibition of OMV secretion reduced it. Purified outer membrane vesicles (OMVs) isolated from the culture supernatant of ATCC 25285 also alleviated DSS-induced goblet-cell depletion when administered intraperitoneally or orally. These findings suggest that ATCC 25285-derived OMVs contribute to the preservation or recovery of goblet-cell numbers under the conditions of this model, although direct promotion of goblet-cell regeneration was not demonstrated [52]. These experiments used ATCC 25285-derived OMVs as a distinct vesicular preparation rather than purified PSA or TP2. The cited study identified microRNA-5119 (miR-5119) as an OMV-associated effector and did not demonstrate that PSA was present in the OMVs or responsible for the observed effects. Therefore, these findings should be attributed to the defined OMV preparation and should not be interpreted as evidence of PSA-mediated activity. A 2026 study extended the evaluation of NCTC 9343-derived outer membrane vesicles to polarized primary human colon-organoid monolayers obtained from three healthy donors. Organoids were exposed apically for 1 h to outer membrane vesicles derived from wild-type NCTC 9343, ETBF, an isogenic ETBF Δ*bft-2* mutant, or an engineered NCTC 9343 strain expressing *bft-2*, at 2 × 10^10^ vesicles/mL, and selected endpoints were evaluated immediately and 24 h after exposure. Under these conditions, the tested vesicle preparations did not significantly alter transepithelial resistance, E-cadherin or claudin-2 abundance, lactate-dehydrogenase release, sodium transport, or the secretion of 48 measured cytokines. However, several vesicle preparations reduced forskolin-stimulated cystic fibrosis transmembrane conductance regulator (CFTR)-mediated chloride secretion. Expression of *bft-2* in the NCTC 9343 background increased this inhibitory effect relative to wild-type NCTC 9343-derived vesicles, whereas deletion of *bft-2* from ETBF did not significantly eliminate the ETBF-associated effect. These results indicate that the contribution of BFT-2 to vesicle-mediated epithelial ion transport is strain-dependent and that additional outer-membrane-vesicle components may contribute. This functional effect on chloride transport should not be interpreted as evidence of improved epithelial-barrier integrity or anti-inflammatory activity. The study was limited to an in vitro organoid model, three healthy donors, and a short exposure period [78]. Microbiota-derived propionate has been shown to stimulate MUC2 expression and mucin production in intestinal goblet cells through a β-oxidation-like pathway that induces hypoxia and activates hypoxia-inducible factor 2 alpha (HIF-2α) [79]. In male C57BL/6J mice, oral administration of a cell-free, concentrated, and pH-adjusted culture supernatant derived from *Lactobacillus rhamnosus* GG ATCC 53103 increased colonic goblet-cell numbers and the expression of MUC2 and 5-hydroxytryptamine receptor 4 (5-HT4R). Pharmacological inhibition of 5-HT4R attenuated the associated goblet-cell secretory response, while separate microbiota analyses showed changes in community composition. However, the study did not establish that microbiota remodeling mediated the increase in mucin production, and direct causal evidence for 5-HT4R remained incomplete [80]. These findings from other probiotic species provide general biological context but do not establish that an equivalent metabolite-mediated or 5-HT4R-dependent pathway operates in NTBF.

Beneath the mucus layer, intestinal epithelial cells strictly control the paracellular permeability of substances through apical tight junctions (TJs) [81]. TJs are composed of multiple protein complexes, including occludin, claudins, and the scaffolding protein zona occludens-1 (ZO-1), among others [82]. In a radiation-induced intestinal injury model, oral administration of live *Bacteroides fragilis* ZY-312 at 1 × 10^9^ CFU per mouse promoted intestinal epithelial proliferation, stem-cell-associated regeneration, mucus secretion, and the recovery of tight-junction-associated proteins, including claudin-1 and ZO-1. ZY-312 increased STAT3 phosphorylation predominantly in small-intestinal epithelial cells. Its protective effects and the restoration of proliferating cell nuclear antigen (PCNA), MUC2, leucine-rich repeat-containing G-protein-coupled receptor 5 (Lgr5), claudin-1, and ZO-1 were markedly attenuated in mice with intestinal-epithelial-cell-specific STAT3 deficiency, indicating that the barrier-associated effects of live ZY-312 were at least partly dependent on epithelial STAT3 signaling under the conditions of this model [53]. In a DSS-induced acute colitis mouse model, oral administration of live *B. fragilis* ATCC 25285, but not a hyperbaric-inactivated preparation, alleviated colitis; increased colonic ZO-1, occludin, and claudin-1 expression; and reduced intestinal permeability. In a separate TNF-α-stimulated hcoEPIC cell model, live ATCC 25285 showed a nonsignificant trend toward reducing apoptosis. Thus, the study did not directly demonstrate that tight-junction restoration was mediated by inhibition of epithelial-cell apoptosis [49]. Changes in tight-junction-associated protein expression represent barrier-related endpoints. Such changes should not by themselves be described as a demonstrated mechanism unless they are accompanied by functional permeability measurements and causal pathway intervention [83].

**Figure 3:**
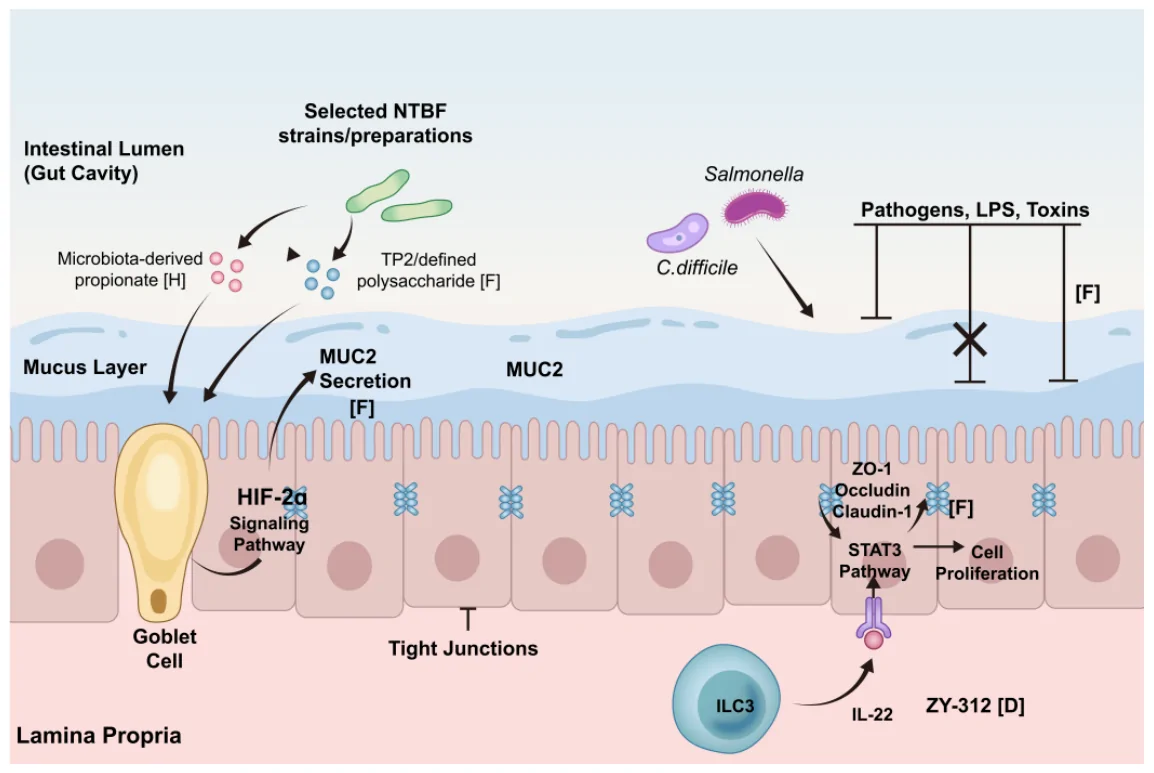
Barrier-associated effects reported for live ZY-312, live ATCC 25285, ZY-312-derived TP2, and ATCC 25285-derived preparations [16,40,47,49,52,53,54,87]. The propionate–HIF-2α pathway is contextual microbiome evidence and has not been established as an NTBF-specific mechanism [79]. Abbreviations: NTBF, nontoxigenic *Bacteroides fragilis*; TP2, a ZY-312-derived zwitterionic capsular polysaccharide preparation; LPS, lipopolysaccharide; MUC2, mucin 2; HIF-2α, hypoxia-inducible factor 2 alpha; ZO-1, zonula occludens-1; STAT3, signal transducer and activator of transcription 3; ILC3, group 3 innate lymphoid cell; IL-22, interleukin-22; ATCC, American Type Culture Collection; *C. difficile*, *Clostridioides difficile*. Evidence labels: [D], direct causal evidence; [F], functional or associative evidence; [H], hypothetical or contextual evidence. Arrows indicate activation or promotion, T-shaped blunt-ended lines indicate inhibition or negative regulation, and the “×” symbol indicates blockade of the corresponding pathway, interaction, or translocation.

Once the integrity of the intestinal barrier function is compromised, it becomes the starting point for multiple disease processes [84,85]. Defects in the intestinal barrier can lead to the translocation of pathogenic microorganisms and their toxins into the lamina propria and even systemic circulation, thereby triggering local or systemic inflammatory responses [86]. NTBF, through the combined effects of enhancing the mucus layer and tight junctions as described above, can effectively improve the intestine’s ability to resist such invasions. In terms of defending against specific pathogens, NTBF exhibits significant protective effects. *Clostridium difficile* is the primary pathogen responsible for antibiotic-associated diarrhea and pseudomembranous colitis, and its pathogenesis is closely associated with gut microbiota dysbiosis and barrier damage. In an antibiotic-disrupted C57BL/6 mouse model, prophylactic oral administration of live *B. fragilis* ZY-312 reduced the morbidity and mortality associated with challenge by *C. difficile* VPI 10463 spores. This protection was accompanied by preservation of intestinal Muc2 and ZO-1 expression and partial restoration of cecal microbial-community composition [40]. In a Caco-2/HT29-MTX/M-like-cell triple co-culture model, exposure to live nontoxigenic *B. fragilis* ATCC 25285 did not significantly alter transepithelial electrical resistance (TEER), Lucifer-yellow permeability, or ZO-1 and occludin messenger RNA (mRNA) expression. The strain also exhibited a very low translocation rate across the epithelial model, supporting its short-term in vitro compatibility with epithelial-barrier integrity under the tested conditions [87]. Live nontoxigenic *B. fragilis* ATCC 25285 and its cell-free culture supernatant reduced S. Heidelberg translocation in an intestinal triple co-culture model without significantly impairing epithelial-barrier integrity or inhibiting pathogen growth, indicating that the effect was not attributable simply to direct growth inhibition [87]. In an antibiotic-associated diarrhea model in Sprague–Dawley rats, oral administration of live NTBF strain ZY-312 improved diarrhea-related outcomes and multiple barrier-associated endpoints, including goblet-cell numbers, MUC2, ZO-1, occludin, aquaporin expression, Ki67-positive epithelial cells, and extracellular signal-regulated kinase (ERK) phosphorylation. These effects were strain-, dose-, and model-dependent and should not be interpreted as uniform properties of NTBF or as evidence of histological structural repair [54]. Furthermore, studies have indicated that bioactive fractions (F3 and F4) derived from the cell-free supernatant of *Bacteroides fragilis* NTBF ATCC 25285 can reduce *Salmonella Heidelberg* virulence. In a BALB/c mouse model, oral administration of these fractions significantly decreased the bacterial loads in Peyer’s patches and spleen, downregulated the expression of virulence genes (*sipA* and *fliC*), attenuated systemic inflammatory cytokines (IL-1β, IL-6, interferon gamma (IFN-γ)), and reduced neutrophil infiltration, without altering overall gut microbiota diversity [15]. NTBF also plays a crucial role in reducing the translocation of harmful substances such as endotoxins. When the intestinal barrier is compromised, macromolecular substances such as bacterial endotoxin lipopolysaccharide (LPS) more readily enter the systemic circulation, triggering inflammation, which is associated with various chronic diseases such as metabolic syndrome and autoimmune diseases [88,89]. NTBF enhances the sealing between epithelial cells by upregulating the expression of tight junction proteins, thereby effectively reducing the passive translocation of these toxins [53]. In summary, selected live NTBF strains and strain-derived preparations have improved mucus- and tight-junction-associated endpoints in specific intestinal-injury models. Causal evidence for epithelial repair is strongest for IL-22–STAT3-associated responses induced by live ZY-312, whereas goblet-cell preservation, MUC2 expression, tight-junction-protein changes, and reduced pathogen translocation observed with other preparations should be interpreted as preparation- and model-specific functional outcomes rather than mechanisms uniformly established across NTBF strains. The barrier-associated effects reported for defined NTBF strains and strain-derived preparations are summarized in Figure 3.

### 4.3. Shaping the Intestinal Environment

Evidence for NTBF-associated microbial competition varies substantially in causal strength. Direct mechanistic evidence supports fragipain-activated antibacterial protoxin 1 (Fab1)-mediated antagonism and T6SS-dependent intraspecific competition in defined *Bacteroides fragilis* strains. By contrast, nutrient depletion, promotion of beneficial taxa, exclusion of unrelated pathogens, and maintenance of community homeostasis remain proposed ecological explanations unless they are supported by defined nutrient-manipulation experiments, functional competition assays, genetic disruption, or rescue experiments. Changes in the relative abundance of microbial taxa following administration of ZY-312 or ZY-312-derived TP2 should therefore be classified as associated community-level changes rather than direct evidence of the mechanisms responsible for those changes. In an antibiotic-associated diarrhea model in Sprague–Dawley rats, oral administration of live NTBF strain ZY-312 at 10^9^ CFU/day significantly increased the fecal relative abundance of *Akkermansia* and decreased that of *Escherichia* on day 17. These findings indicate selective modulation of specific microbial taxa rather than complete restoration of the overall gut microbial community [54]. In an in vitro simulated colonic fermentation system, ZY-312-derived TP2 altered microbial community composition in a concentration-dependent manner, including changes in the abundance of *Faecalibacterium*, Romboutsia, Ruminococcaceae, and several Proteobacteria. However, TP2 did not significantly change SCFA concentrations, including butyrate, in the same system [90]. In conventional SPF C57BL/6J mice, live nontoxigenic *B. fragilis* NCTC 9343 predominantly colonized the cecum and colon, with relatively low abundance in the small intestine. Repeated oral administration markedly increased its cecal abundance without producing major detectable changes in the overall composition of the pre-existing gut microbiota [44]. These findings support region-specific intestinal persistence during repeated administration. However, they do not by themselves establish direct mucosal association, ecological engraftment after treatment withdrawal, or permanent colonization. This distribution pattern may facilitate local ecological and host–microbe interactions but should not be regarded as a prerequisite for all NTBF-associated effects.

**Figure 4:**
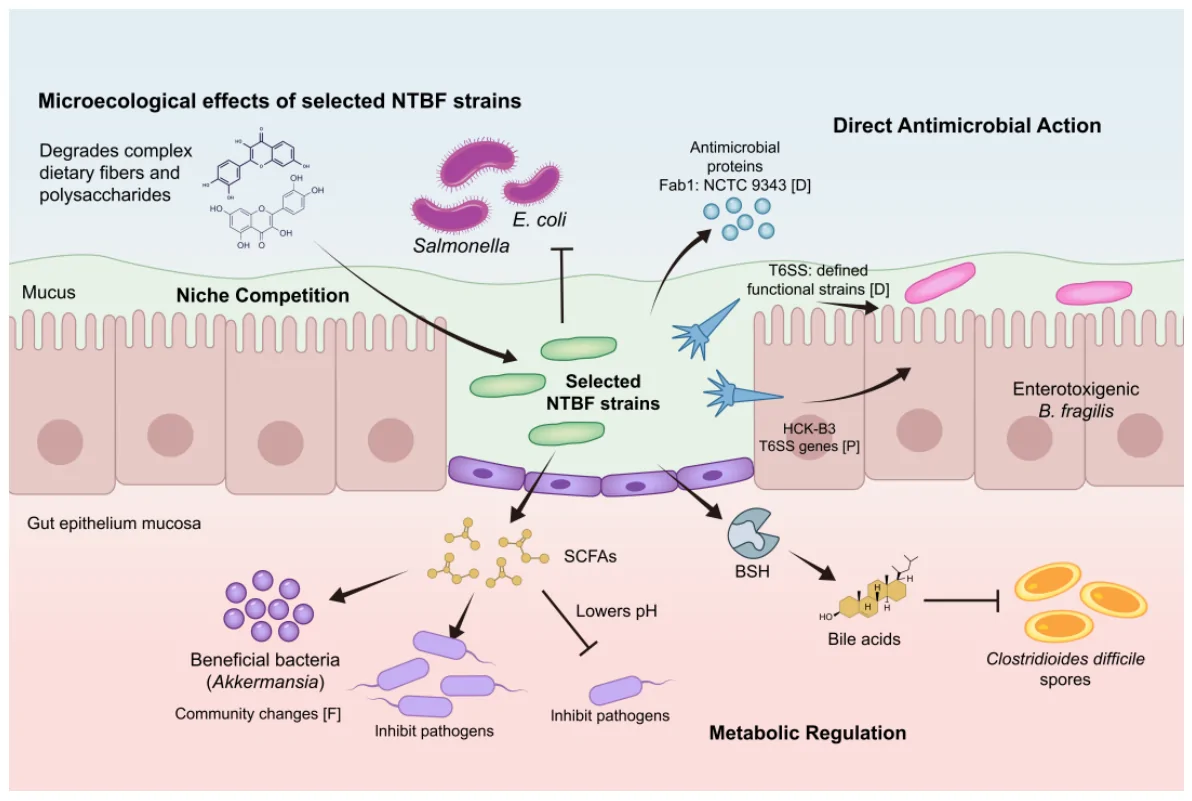
Microecological effects reported across selected NTBF strains. Direct evidence includes NCTC 9343-derived Fab1 and T6SS-dependent competition in defined strains, whereas microbiota changes, nutrient competition, SCFA-related effects, and bile-acid pathways have functional, predicted, or contextual evidence of varying strength [18,21,35,54,55,87,90,91,92]. Abbreviations: NTBF, nontoxigenic *Bacteroides fragilis*; NCTC, National Collection of Type Cultures; Fab1, a fragipain-activated antibacterial protoxin produced by NCTC 9343; T6SS, type VI secretion system; SCFAs, short-chain fatty acids; BSH, bile salt hydrolase; *E. coli*, *Escherichia coli*; *B. fragilis*, *Bacteroides fragilis*; *C. difficile*, *Clostridioides difficile*. Evidence labels: [D], direct causal evidence; [F], functional or associative evidence; [P], genome-predicted and unverified feature; [H], hypothetical or contextual evidence. Arrows indicate activation or promotion, T-shaped blunt-ended lines indicate inhibition or negative regulation.

In addition to passively competing for nutrients, NTBF can also secrete various antimicrobial substances that directly inhibit or kill other competitors. These substances mainly include bacteriocins and antimicrobial metabolites. Bacteriocins are ribosomally synthesized proteins or peptides with bactericidal or bacteriostatic activity, typically exhibiting high specificity against strains closely related to the producing bacterium, thereby efficiently eliminating such strains [18]. Functional T6SSs can mediate contact-dependent intraspecific competition in defined *Bacteroides fragilis* strains. However, T6SS gene content and activity vary among strains, and this mechanism should not be considered a general property of NTBF. Although the specific molecular characteristics of the bacteriocins produced by NTBF are still under in-depth investigation, there is clear evidence indicating that it possesses this capability. The nontoxigenic *B. fragilis* strain NCTC 9343 produces Fab1, an antibacterial protoxin that is cleaved and activated by the conserved cysteine protease fragipain. Activated Fab1 kills susceptible *B. fragilis* strains and delayed invasion by the Fab1-sensitive strain 638R in gnotobiotic mice. Because fab1 is heterogeneously distributed among *B. fragilis* strains, this represents a strain-specific competitive mechanism rather than a general property of NTBF [91]. Certain *Bacteroides fragilis* strains possess bile salt hydrolase activity, which deconjugates conjugated primary bile acids and may thereby contribute to their subsequent microbial conversion into secondary bile acids. Secondary bile acids such as deoxycholic acid can restrict the vegetative growth of *Clostridioides difficile*. However, the cited evidence does not establish that a specific NTBF strain alone produces the full range of inhibitory secondary bile acids. Separately, an in vitro study summarized in this review reported that co-incubation with an NTBF preparation reduced *C. difficile* biofilm formation, although the specific strain and experimental preparation should be verified from the primary study [17]. In summary, NTBF may shape the intestinal microecological environment through efficient nutrient competition, strain-specific antagonistic mechanisms such as bacteriocin production, and changes in nutrient availability and community-level metabolite profiles. These effects may restrict pathogen colonization and contribute to microbial-community stability. Collectively, selected NTBF strains can influence microbial competition or community composition through strain-specific antagonistic systems and ecological interactions. However, the relative contributions of T6SS activity, bacteriocin production, nutrient competition, community remodeling, and metabolite exchange remain incompletely resolved and should not be generalized across NTBF strains. This capacity to actively shape the intestinal environment serves as an important ecological foundation for the realization of its beneficial functions. The strain-specific microecological effects and their different levels of supporting evidence are summarized in Figure 4.

In a vertical-transmission model involving the developing microbiota of C57BL/6 mice, early administration of a live T6SS-competent NTBF strain reduced ETBF colonization, whereas later administration or disruption of the NTBF T6SS weakened this competitive effect. Catalytically active BFT enabled ETBF to access a lamina propria niche through a developmentally restricted, goblet-cell-associated process; wild-type NTBF remained luminally restricted, while engineered expression of active BFT conferred lamina propria access. Thus, the study supports temporally and genetically constrained strain competition and BFT-dependent niche acquisition but does not directly demonstrate competition through nutrient limitation, priority effects, or niche saturation [92]. In animal models of sterile or simplified communities, pre-colonized NTBF did show the ability to reduce the success rate of subsequent ETBF colonization, which was particularly evident in intraspecific exclusion. However, this rejection effect has strong strain specificity, and not all NTBF strains have the same competitive ability or produce antibacterial factors that inhibit other strains of the same species [17]. NTBF may exert disease-promoting effects under specific metabolic and host conditions. In high-fat-diet-fed Cdx2Apc^f/w^ mice, colonization with a live *B. fragilis* 638R strain engineered to express the NCTC 9343-derived bile salt hydrolase (BSH) gene increased colonic unconjugated bile acids and was associated with greater tumor burden and enhanced WNT/β-catenin-associated signaling compared with an isogenic low-BSH 638R control. Pharmacological BSH inhibition attenuated these effects, supporting a BSH-dependent contribution under this specific model rather than a general tumor-promoting property of NTBF [55]. The biological effects of NTBF cannot be predicted solely from its abundance or non-toxigenic status. Under specific dietary and genetic conditions, a high-BSH NTBF preparation was associated with increased colorectal tumor progression, although cooperation with ETBF was not examined [55]. Separately, pattern I ETBF and pattern II or III NTBF isolates obtained from colorectal biopsy specimens were all capable of forming biofilms in vitro, with significantly greater biofilm formation observed among ETBF isolates. However, this study did not demonstrate mixed NTBF–ETBF biofilms in tumor tissue or a causal role for biofilm formation in CRC progression [93].

## 5. Multifunctional Contribution of Beneficial Metabolites

NTBF may influence host physiology through both strain-derived products and changes in community-level metabolite profiles. These effects may involve direct bacterial metabolites as well as indirect metabolic changes resulting from microbial-community remodeling. Among gut microbial metabolites, short-chain fatty acids (SCFAs) are well characterized, but their sources and relative contributions should be distinguished at the strain and community levels. Short-chain fatty acids (SCFAs) are major products of dietary fiber fermentation by the gut microbiota and mainly include acetate, propionate, and butyrate [19]. Acetate is the most abundant SCFA in the colonic lumen and is produced by diverse members of the gut microbiota. After absorption, acetate can contribute to peripheral lipid and energy metabolism [94,95]. In male ICR mice with acute hyperuricemia induced by 10% fructose in drinking water and intraperitoneal potassium oxonate, oral administration of live *Bacteroides fragilis* ATCC 25285 at 1 × 10^10^ CFU/day for 7 days reduced plasma uric acid and was accompanied by increased fecal succinate and propionate levels. Because these metabolites were measured in feces from conventionally colonized mice rather than in a defined culture or isotope-tracing system, the increased fecal propionate and succinate levels cannot be attributed exclusively to direct production by ATCC 25285. In separate experiments, oral sodium succinate reduced plasma uric acid and inhibited hepatic adenosine monophosphate deaminase 2 (AMPD2)-associated purine metabolism, supporting a functional role for succinate but not proving that all effects of live ATCC 25285 were mediated exclusively by succinate [56]. Among microbiota-derived SCFAs, butyrate is an important energy substrate for colonocytes and has well-established roles in epithelial-barrier maintenance, histone deacetylase inhibition, and immune regulation. However, intestinal butyrate is produced predominantly by other bacterial taxa, particularly members of the Lachnospiraceae and Ruminococcaceae families, rather than by *B. fragilis*. Therefore, the general physiological functions of butyrate should not be interpreted as evidence of direct butyrate production by NTBF [96,97]. In addition to organic-acid-associated effects, selected *Bacteroides fragilis* strains produce or contain strain-specific bioactive components. Among the best-characterized strain-derived products, purified canonical PSA from *Bacteroides fragilis* NCTC 9343 has demonstrated immunomodulatory activity in defined experimental systems. This evidence should not be attributed to TP2, total capsular-polysaccharide preparations, OMVs, cell-free culture supernatants, or intact bacteria unless PSA dependence was directly demonstrated. However, PSA production and activity have not been demonstrated in all NTBF strains, and the effects of intact bacteria cannot be attributed exclusively to PSA. Other *B. fragilis* strains may produce structurally distinct extracellular polysaccharides, but their identities and biological activities require strain-specific characterization. Activities reported for extracellular polysaccharides from other probiotic species should not be directly extrapolated to NTBF-derived polysaccharides [98]. As an example of the functional diversity of strain-derived products, the filtered cell-free culture supernatant and intracellular cell-free extract of the nontoxigenic *Bacteroides fragilis* strain SNBF-1 reduced lipid accumulation and altered lipid-related parameters in fatty-acid-challenged HepG2 cells. These preparations also increased glucose consumption and glycogen content in an insulin-resistant HepG2 cell model and exhibited radical-scavenging activity in cell-free antioxidant assays. However, the specific extracellular polysaccharides or other bioactive components responsible for these effects were not identified [21]. These findings suggest that SNBF-1-derived extracellular and intracellular components possess multiple metabolic activities in vitro, although their chemical identities and contributions remain to be determined. These findings demonstrate multiple in vitro activities of SNBF-1-derived extracellular and intracellular preparations, but the responsible components and their relevance to intestinal microbial ecology remain unresolved. Fermentation of chicory with *B. fragilis* DSM1396 generated a preparation containing filtered fermentation products and heat-inactivated bacterial biomass (C-*B. fragilis*) and a separate filtered phyto-postbiotic supernatant (PPS). In vitro, PPS reduced lipid accumulation during human adipocyte differentiation, whereas C-*B. fragilis* decreased leptin mRNA expression in human adipospheroids without significantly altering leptin secretion. These findings indicate that DSM1396-derived chicory fermentation products possess preliminary metabolic activity, although the responsible bioactive components and their in vivo relevance remain unresolved [99]. These findings provide preliminary evidence of metabolic activity in human adipocyte models in vitro, but they do not establish effects on whole-body energy balance or obesity-related disease progression in vivo. These metabolic effects should be interpreted at both the strain and microbial-community levels. Strain-derived products such as PSA may act directly on host cells, whereas changes in SCFA profiles may reflect both bacterial metabolism and broader community remodeling. Future studies should therefore distinguish direct strain-derived metabolites from community-level metabolic changes using strain-resolved metabolomics, isotope tracing, and defined microbial consortia, while further clarifying their biosynthetic pathways, molecular targets, and dose–response relationships.

## 6. The Application and Potential of NTBF in Disease Models

Preclinical studies have evaluated selected NTBF strains and strain-derived preparations in models of inflammatory bowel disease, necrotizing enterocolitis, antibiotic-associated diarrhea, metabolic disorders, and colorectal tumorigenesis. The strength and nature of the evidence vary substantially according to the strain, experimental preparation, host, disease model, and timing of administration. In colorectal cancer-related studies, direct prophylactic evidence is currently limited to defined experimental settings in which a specific live NTBF strain was administered before tumor-promoting bacterial challenge. Findings based only on reduced inflammation, altered ETBF abundance, microbial-community changes, or tumor-associated biomarkers should be considered preliminary or indirect rather than evidence of colorectal cancer prevention. For disease-oriented studies, evidence was classified according to model type, causal strength, and translational proximity. Category A comprises in vivo studies incorporating causal pathway interruption, knockout models, isogenic-strain comparisons, or intervention-timing controls. Category B comprises in vivo functional-efficacy studies supported mainly by clinical, histological, immunological, barrier-associated, or microbiome endpoints without complete causal validation. Category C comprises cell-line studies and human observational associations, which may provide mechanistic or clinical-contextual information but do not constitute in vivo intervention evidence. Table 2 therefore summarizes representative Category A and Category B in vivo studies only. Category C evidence is discussed separately in the corresponding narrative sections and is not placed at the same evidentiary level as the animal intervention studies.

IBD is a chronic, relapsing inflammatory disease of the gastrointestinal tract involving complex interactions among genetics, immunity, environment, and the gut microbiota [100]. Selected NTBF strains and strain-derived preparations have shown anti-inflammatory and barrier-associated effects in specific experimental models of intestinal inflammation. The strength of mechanistic evidence varies among studies: knockout, pathway-intervention, or purified-molecule experiments provide direct causal evidence, whereas changes in cytokines, signaling proteins, histopathology, barrier-associated markers, or microbial composition provide functional or indirect support unless the responsible pathway was directly tested. Studies on the ZY-312 strain have revealed its detailed protective mechanism in a DSS-induced mouse model of colitis. Oral administration of live ZY-312 significantly reduced the disease activity index, ameliorated colon shortening, and improved histopathological damage. Further mechanistic studies indicated that its protective effect is achieved through activation of the group 3 innate lymphoid cell (ILC3)–IL-22–STAT3 signaling axis: ZY-312 promoted the activation of IL-22-secreting group 3 innate lymphoid cells (ILC3s) in the gut, thereby increasing IL-22 production; IL-22 then activated the STAT3 signaling pathway in intestinal epithelial cells, ultimately promoting colonic mucosal regeneration and repair [16]. Furthermore, in a TNBS-induced colitis model in male Wistar rats, treatment with purified capsular polysaccharide TP2 derived from *Bacteroides fragilis* ZY-312 improved disease activity and reduced colonic ulceration and histopathological damage. TP2 also improved mucus barrier-related indicators and tight junction protein expression, modulated the levels of both pro-inflammatory cytokines and anti-inflammatory cytokines, and partially reshaped the dysbiotic gut microbiota [47]. IBD animal models are key tools for studying its pathology and treatment [101]. Collectively, selected NTBF strains or strain-derived preparations have modulated immune, barrier-associated, and microbial endpoints in specific experimental colitis models [16,47,49].

Necrotizing enterocolitis (NEC) is a severe and potentially life-threatening gastrointestinal disease in newborns, especially preterm infants, characterized by intestinal inflammation, ischemic necrosis, and bacterial translocation [102]. The pathogenesis of NEC is closely associated with intestinal immaturity, enteral feeding challenges, and gut microbiota dysbiosis. In the pathological process of NEC, gut microbiota dysbiosis leads to excessive activation of the TLR4 pathway, triggering a severe inflammatory cascade, and is considered one of the core driving factors [103]. Therefore, modulating the gut microbiota and suppressing inflammation through probiotics are considered promising preventive strategies [104]. In a neonatal rat model of necrotizing enterocolitis induced by *Cronobacter sakazakii* challenge and repeated hypoxic stress, pretreatment with live *Bacteroides fragilis* ZY-312 reduced mortality, intestinal injury, intestinal permeability, and the loss of ileal ZO-1 expression. Treatment also moderated changes in TNF, IFN-γ, and IL-10 and reduced markers associated with epithelial apoptosis and pyroptosis [48]. These outcomes provide preparation- and model-specific functional evidence for the protective effect of live ZY-312 in this neonatal-rat model. In addition, this experimental model combined a defined enteric pathogen with repeated hypoxic stress in neonatal rats and therefore does not reproduce the full developmental, nutritional, microbial, and clinical heterogeneity of NEC in preterm human infants. ZY-312 was administered prophylactically before pathogen challenge, and reproducibility across independent laboratories, additional strains, different doses, and therapeutic intervention windows has not yet been established.

Antibiotic-associated diarrhea (AAD) is one of the most common side effects of antibiotic therapy, and its root cause is the disruption of gut microbiota balance by broad-spectrum antibiotics [105]. Live ZY-312 has been evaluated in antibiotic-associated diarrhea and *Clostridioides difficile* infection models, with outcomes that differed according to the host species, antibiotic regimen, administered dose, and whether treatment was therapeutic or prophylactic. In a Sprague–Dawley rat model of antibiotic-associated diarrhea induced by oral administration of clindamycin, ampicillin, and streptomycin, treatment with live *Bacteroides fragilis* ZY-312 improved diarrhea-related outcomes and was accompanied by improvements in mucus barrier function, tight junction-associated proteins, aquaporin expression, and epithelial proliferative responses [54]. *Clostridium difficile* infection is an important cause of severe antibiotic-associated diarrhea and colitis. In an antibiotic-disrupted male C57BL/6 mouse model challenged with *C. difficile* VPI 10463 spores, prophylactic oral administration of live ZY-312 at 1 × 10^7^ or 1 × 10^8^ CFU/day markedly increased survival and improved diarrhea-related clinical manifestations. Treatment also reduced cecal and colonic histopathological damage, preserved MUC2 and ZO-1 expression, and partially improved cecal microbial diversity and community composition. The relative abundance of *Akkermansia muciniphila* was positively associated with ZY-312 treatment, although its causal contribution to protection was not established [40]. The gut microbiota is closely related to host metabolism, and NTBF and its metabolites play a role in regulating metabolism and inflammatory responses. Studies have shown that NTBF is associated with improving metabolic profiles and alleviating inflammatory responses in obesity and diabetes models. Evidence for direct NTBF-mediated metabolic regulation remains limited. Reported changes in host metabolic outcomes, inflammatory markers, microbial composition, or fecal metabolites should be classified as functional or indirect evidence unless the responsible strain-derived metabolite and causal host pathway are directly validated.

Among them, ETBF has been confirmed to be highly associated with IBD and CRC. The BFT toxin it secretes can induce cleavage of E-cadherin, trigger colonic inflammation, and promote colon tumorigenesis through IL-17-dependent inflammatory pathways [106]. In specific-pathogen-free C57BL/6 wild-type and MinApc716+/− mice, prior colonization with live nontoxigenic *B. fragilis* NCTC 9343 before challenge with enterotoxigenic *B. fragilis* strain 86-5443-2-2 reduced chronic colitis and tumor development. This protection was associated with the dominance of NCTC 9343 and reduced interleukin-17A (IL-17A) responses. Similar prophylactic effects were observed with an isogenic PSA-deficient NCTC 9343 mutant, indicating that protection in this model was not dependent on PSA. However, simultaneous administration of the two strains or administration of NCTC 9343 after stable ETBF colonization did not provide comparable protection. These findings indicate that the protective effect of NCTC 9343 was prophylactic and strongly dependent on the timing and order of bacterial colonization [73]. Human clinical evidence remains limited and primarily associative. In a retrospective study of 197 patients with pathological stage II or III colorectal cancer who underwent curative resection, *Bacteroides fragilis* 16S ribosomal RNA (16S rRNA) deoxyribonucleic acid (DNA) was detected in cancerous or adjacent non-cancerous tissues from 120 patients. Among these patients, 108 were classified as NTBF-positive because *bft* DNA was not detected, whereas 12 were classified as ETBF-positive. Although the presence of *B. fragilis* was associated with a favorable recurrence-free-survival estimate in multivariable analysis, no statistically significant differences in recurrence-free or overall survival were observed between the NTBF-positive and ETBF-positive groups. Because the study detected bacterial DNA in formalin-fixed tissues without isolating or characterizing individual strains, it does not establish strain-specific causality, probiotic activity, or safety [107]. Thus, the available evidence supports a prophylactic effect of live NCTC 9343 in a specific ETBF-associated chronic colitis and tumorigenesis model. It does not establish a general colorectal-cancer-preventive effect of NTBF or therapeutic efficacy after stable ETBF colonization. Collectively, selected NTBF strains and strain-derived preparations have shown beneficial effects in specific preclinical disease models. The evidence remains uneven across strains and preparations, and no uniform therapeutic indication can currently be assigned to NTBF as an entire category.

Across the disease areas reviewed above, the evidence remains heterogeneous in model type, causal strength, and translational proximity. Most findings derive from individual studies conducted with a single strain or preparation, relatively small animal cohorts, short experimental periods, and prophylactic or early-intervention designs. Dose–response relationships were evaluated in only a subset of studies, and independent replication across laboratories was generally limited. Chemical colitis, neonatal pathogen–hypoxia NEC, antibiotic-perturbed infection, and genetically predisposed or bacteria-driven tumor models capture different components of human disease and should not be interpreted as interchangeable evidence. The studies included in this review do not establish clinical efficacy of a defined NTBF strain in patients with IBD, NEC, ADD, *Clostridioides difficile* infection (CDI), or colorectal cancer.

## 7. Challenges and Prospects

NTBF shows great application potential as a next-generation probiotic, yet its clinical translation faces multiple challenges, mainly in areas such as standardized cultivation, delivery technology, host heterogeneity, and long-term safety [12]. These challenges primarily center on the difficulties in production and delivery due to its strict anaerobic nature, the impact of substantial inter-individual host heterogeneity on efficacy consistency, and the long-term safety validation and precise efficacy assessment that must be completed before clinical application. Acknowledging and addressing these challenges, while seizing opportunities from new technological developments, is key to realizing the clinical value of NTBF. *B. fragilis* is an obligate anaerobe that is extremely sensitive to oxygen, which not only complicates laboratory research but also constitutes the primary technical obstacle to industrial-scale production. Strict anaerobic cultivation requires creating and maintaining a reducing environment with a low redox potential, which is not simply a matter of removing oxygen. It typically necessitates the addition of reducing agents such as sodium thioglycolate, cysteine, or hemin to the culture medium, which not only consume dissolved oxygen but also serve as electron donors or buffers to maintain the reducing state of the medium [108]. These stringent process requirements and the dependence on specialized medium components significantly increase the complexity of production, the difficulty of control, and the overall cost. As a live microbial product, orally administered NTBF should reach the relevant intestinal region in sufficient numbers and retain adequate viability and functional activity during gastrointestinal transit. Stable colonization may enhance or prolong some strain-specific effects, particularly those involving sustained ecological competition or repeated host–microbe interactions, but it is not necessarily required for all probiotic or live-biotherapeutic activities. Transient passage may also permit interactions with epithelial and immune cells or exposure to strain-derived metabolites and other bioactive products. For clarity, gastrointestinal-transit survival refers to the maintenance of viability and functional activity during passage through the gastrointestinal tract; temporary residence refers to short-term strain detection during or shortly after administration; mucosal association refers to attachment to or close association with the mucus layer or mucosal surface; ecological engraftment refers to sustained incorporation into the resident microbial community; and long-term colonization refers to strain-resolved persistence after administration has ceased. These states are not interchangeable, and detection during repeated administration does not by itself demonstrate ecological engraftment or long-term colonization. The reviewed studies did not evaluate these stages uniformly. Most NTBF disease-model studies reported the administration schedule and disease-related outcomes but did not perform strain-resolved anatomical recovery, direct mucosal-association analysis, or prolonged follow-up after treatment withdrawal. Therefore, such studies are not described as demonstrating ecological engraftment or long-term colonization unless these endpoints were directly assessed. Oral delivery nevertheless remains challenging because NTBF must tolerate gastric acidity, bile salts, and digestive enzymes to reach the lower intestine in a functionally active state [109]. Traditional probiotic delivery technologies, such as spray drying and freeze-drying, have limitations in protecting oxygen-sensitive NTBF, often leading to a substantial reduction in viable cell counts during processing and storage. Furthermore, the resident gut microbiota already occupies many ecological niches and may limit the survival, persistence, or stable colonization of an administered strain. Host factors such as genotype, dietary composition, baseline microbiota profile, immune status, and sex may influence NTBF survival, transient persistence, host interactions, and colonization, thereby contributing to inter-individual variability in efficacy [110]. The significant effects demonstrated by NTBF in a particular animal model may not necessarily be replicated in broader clinical applications. For example, an individual’s resident gut microbiota, immune response status, and intestinal permeability can profoundly influence the survival of NTBF, its interaction with the host, and the ultimate health outcomes. Therefore, pursuing universal efficacy of a single strain while ignoring host heterogeneity is likely to lead to inconsistent clinical trial results, although preliminary safety assessments have provided safety data for NTBF strains [22,35]. In a Caco-2 cell model, overnight treatment with heat-inactivated nontoxigenic *Bacteroides fragilis* ATCC 23745, its filtered cell-free culture supernatant, or purified outer membrane vesicles produced preparation-specific changes in genes associated with the endocannabinoid system and epithelial tight junctions. Heat-inactivated ATCC 23745 increased *tjp1* mRNA expression, outer membrane vesicles increased *tjp1* and *pparg* expression, and the cell-free culture supernatant increased *ocln* and *pparg* expression. In contrast, live ATCC 23745 produced partly different or opposite transcriptional responses. Because the study assessed mRNA expression only and did not directly measure epithelial permeability, tight junction protein abundance, pathogen translocation, in vivo barrier function, or storage stability, these findings indicate potential barrier-related activity rather than demonstrated barrier protection [111]. However, the safety of long-term administration and the potential risks under special physiological or pathological conditions still need to be verified through longer and more rigorous clinical trials. Although NTBF does not produce BFT, as a member of the genus *Bacteroides*, whether certain strains may exhibit other opportunistic pathogenicity under specific circumstances, or whether their metabolic activities may have unexpected effects on host metabolism over the long term, requires ongoing attention and evaluation [17].

In response to the above challenges, future research should move toward technological innovation and systematic evaluation to fully unlock the clinical application potential of NTBF. Overcoming the difficulties in production and delivery depends on technological innovation. In terms of cultivation, optimizing anaerobic fermentation processes and developing novel protective agents and lyophilization techniques are fundamental to improving the yield and stability of live NTBF [108]. In terms of delivery, novel encapsulation technologies that go beyond traditional formulations are emerging. For example, biofilm-based probiotic delivery systems show unique advantages; the biofilm formed by the bacteria themselves provides natural physical protection, significantly enhancing the bacteria’s tolerance to gastric acid, bile salts, and oxygen, thereby increasing the rate of live bacteria reaching the gut after oral administration [112]. Furthermore, encapsulation using microcapsules, liposomes, or natural polysaccharides is also an effective strategy for protecting anaerobic bacteria and achieving colon-targeted release [109]. These delivery systems may improve the survival and intestinal delivery of NTBF, but their effects on viable-dose recovery, biological activity, storage stability, and product consistency require strain- and formulation-specific validation. Artificial intelligence, machine learning, and genome-scale metabolic models may eventually support hypothesis generation, candidate-strain screening, and personalized strain selection. However, their application to NTBF remains exploratory because sufficiently large strain-resolved datasets, prospective predictive validation, and demonstrated clinical utility are currently lacking. These approaches should therefore be regarded as future research possibilities rather than near-term solutions [113]. Future research should fully leverage multi-omics technologies, including metagenomics, transcriptomics, proteomics, and metabolomics, to systematically investigate host and microbial changes associated with NTBF exposure, gastrointestinal transit, temporary residence, mucosal association, ecological engraftment, or long-term colonization [12]. Spatial and strain-resolved methods should be used to determine where viable NTBF is detected, whether it remains luminal or associates with the mucus and mucosal surfaces, and how long it persists after administration has ceased. Future clinical trials need to be scientifically designed, including clearly defined primary and secondary efficacy endpoints, appropriate placebo controls, adequate stratification and subgroup analyses, and long-term safety follow-up. The development of NTBF as a live biotherapeutic product will require a strain-specific and traceable quality-management framework. This framework should include confirmation of strain identity; establishment of master and working cell banks; monitoring of genetic stability, viable-cell counts and biological potency; and control of microbial contamination, bacteriophage risks, residual culture-medium components, storage stability, and batch-to-batch consistency. Clinical development should also evaluate fecal shedding, persistence after administration, potential environmental release, and adverse events during treatment and follow-up. These aspects have not yet been systematically evaluated for the NTBF strains reviewed here and therefore remain priorities for future product development and pharmacovigilance. Synthetic-biology modification of NTBF may be explored in the longer term to introduce defined functions or remove undesirable traits. However, genetically modified NTBF strains would require separate evaluation of genetic stability, biological containment, unintended functional changes, environmental release, and clinical safety and should not be presented as near-term therapeutic solutions. Similarly, combining NTBF with complementary microorganisms represents a future research possibility, and direct testing of strain compatibility, product stability, safety, and efficacy would be required before synergistic effects could be established. In summary, selected NTBF strains have potential for future development as live biotherapeutic candidates. However, their clinical translation will depend on strain-specific dose definition, validated potency and quality-control assays, standardized manufacturing, comprehensive safety assessment, and controlled clinical trials. Artificial intelligence-assisted selection, synthetic biology, and personalized NTBF interventions remain longer-term possibilities that require substantial experimental and clinical validation.

## 8. Summary and Discussion

**Figure 5:**
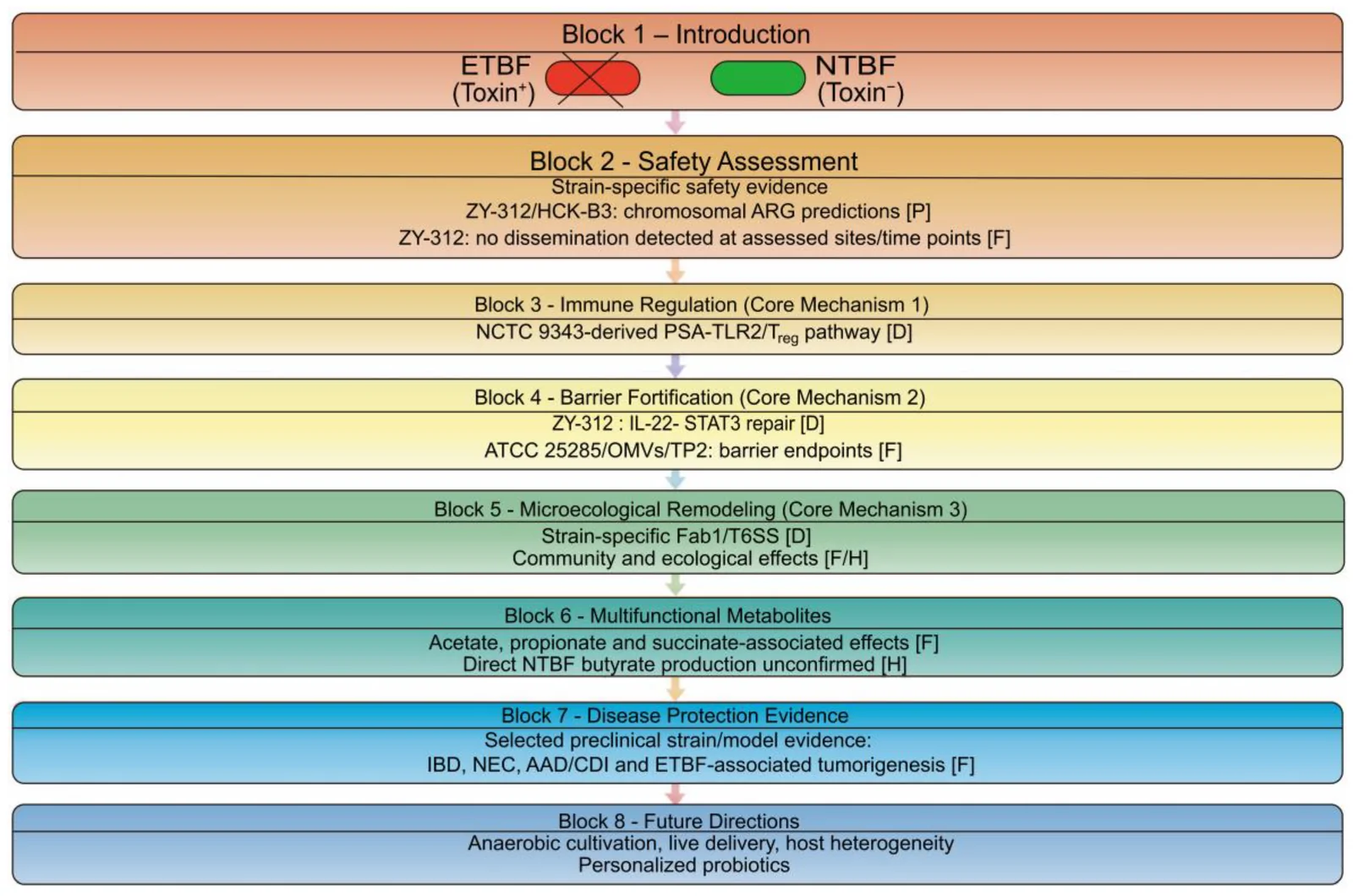
Summary of strain-specific NTBF evidence from biological characterization to future development. The diagram integrates findings from different strains, preparations, hosts, and models and should not be interpreted as a universal or sequential NTBF mechanism [16,22,35,40,43,47,48,53,54,55,73,91,92]. Abbreviations: ETBF, enterotoxigenic *Bacteroides fragilis*; NTBF, nontoxigenic *Bacteroides fragilis*; ARG, antimicrobial-resistance gene; NCTC, National Collection of Type Cultures; PSA, polysaccharide A; TLR2, Toll-like receptor 2; Treg, regulatory T cell; IL-22, interleukin-22; STAT3, signal transducer and activator of transcription 3; ATCC, American Type Culture Collection; OMVs, outer membrane vesicles; TP2, a ZY-312-derived zwitterionic capsular polysaccharide preparation; Fab1, a fragipain-activated antibacterial protoxin; T6SS, type VI secretion system; IBD, inflammatory bowel disease; NEC, necrotizing enterocolitis; AAD, antibiotic-associated diarrhea; CDI, *Clostridioides difficile* infection. Evidence labels: [D], direct causal evidence; [F], functional or associative evidence; [P], genome-predicted and unverified feature; [H], hypothetical or contextual evidence. Toxin+ and Toxin− indicate toxin-producing and non-toxin-producing phenotypes, respectively. The “×” symbol in Block 1 indicates that toxin-producing ETBF is excluded from the NTBF category.

As an important member of the gut microbiota, NTBF stands in stark contrast to the pathogenicity of ETBF and exhibits multifaceted probiotic potential. This review systematically summarizes the biological characteristics, safety profile, core probiotic mechanisms, and applications of NTBF in disease models, aiming to provide a scientific basis for its development as a next-generation beneficial bacterial strain. An integrated summary of the strain-specific evidence and future development considerations is presented in Figure 5.

The absence of the *bft* gene defines the nontoxigenic status of *Bacteroides fragilis* strains and removes a major known virulence determinant of ETBF, but it does not by itself establish comprehensive safety. Current genomic, antimicrobial-susceptibility, biodistribution, and preclinical toxicological evidence supports the preliminary safety or short-term tolerability of selected strains, particularly ZY-312 and HCK-B3, under the specific experimental conditions examined. For these strains, several predicted antimicrobial-resistance genes were chromosomally located rather than plasmid-borne. However, chromosomal localization does not eliminate horizontal-transfer risk, and the mobility of these loci has not been fully resolved through comprehensive flanking-region analysis or experimental transfer assays. No evident toxicity was observed in the evaluated animal models under the specific conditions tested. Studies using ZY-312 did not detect obvious dissemination to the examined peripheral organs at the assessed time points, whereas HCK-B3 showed no evident adverse effects in either healthy mice or cyclophosphamide-induced immunosuppressed mice. However, these findings should not be extrapolated to all NTBF strains, and each candidate strain requires independent assessment of virulence determinants, antimicrobial-resistance phenotypes, resistance-gene flanking regions, genomic islands, transposons, integrative and conjugative elements, insertion sequences, experimental transferability, translocation potential, administered dose, host condition, and long-term exposure.

Mechanistic evidence has been obtained from selected live NTBF strains and defined strain-derived preparations rather than from NTBF as a uniform category. Purified PSA derived mainly from NCTC 9343 has been associated with TLR2-dependent immune regulation, regulatory T-cell responses, IL-10 production, and context-dependent modulation of NF-κB-associated signaling. Live ZY-312 and its purified capsular polysaccharide TP2 have shown barrier-associated and immunomodulatory effects in distinct intestinal injury and colitis models, whereas live ATCC 25285, its outer membrane vesicles, and its cell-free culture supernatant produced preparation-specific effects on inflammatory signaling, goblet-cell preservation, tight junction-associated proteins, and pathogen translocation. Strain-specific competitive systems, including Fab1 production and T6SS-dependent antagonism, have also been demonstrated in selected strains. These immune, barrier-associated, and microecological effects vary according to the strain, experimental preparation, administered dose, host condition, and disease model and should not be regarded as mechanisms uniformly shared by all NTBF strains.

Selected *Bacteroides fragilis* strains have been associated with acetate, propionate, and succinate metabolism, and administration of some strains has altered community-level SCFA profiles. However, these community-level changes do not establish direct metabolite production by the administered strain. Direct butyrate production by NTBF has not been demonstrated, and possible cross-feeding effects require strain-resolved validation. Selected NTBF strains have shown protective effects in specific preclinical models of inflammatory bowel disease, necrotizing enterocolitis, antibiotic-associated diarrhea, and ETBF-associated colorectal tumorigenesis. However, high bile salt hydrolase activity in a defined nontoxigenic *Bacteroides fragilis* preparation promoted colorectal tumor progression under specific dietary and genetic conditions. Thus, the effects of NTBF on colorectal cancer depend on the strain, metabolic activity, host condition, timing of administration, and experimental model and cannot be generalized to NTBF as an entire category [55].

Although NTBF holds great promise, its clinical translation still faces challenges such as difficulties in strict anaerobic cultivation, delivery system hurdles, host heterogeneity, and the need for long-term safety validation. Immediate priorities should include strain-specific manufacturing, dose and potency definition, comprehensive safety assessment, and rigorous clinical trials. Multi-omics and personalized strain-selection approaches may be explored as longer-term research directions but require prospective validation before clinical application. In summary, selected NTBF strains remain potential candidates for future microbiota-based interventions; however, their efficacy and safety must be established through strain-specific manufacturing, mechanistic validation, comprehensive safety assessment, and controlled human trials.

## Figures and Tables

**Table 1:** Strain-level heterogeneity of representative NTBF strains and evidence of their beneficial effects.

Name of Strain	Source	*Bft*	Capsular-Polysaccharide Evidence and Preparation	T6SS	Main Metabolic and Functional Mechanisms	Disease Model
ZY-312 [16,47,48]	Healthy baby faeces	Not directly assessed in these studies	Purified ZY-312-derived zwitterionic capsular polysaccharide TP2 (~70 kDa); direct biochemical equivalence with canonical NCTC 9343 PSA has not been established.	T6SS not characterized; pathogen-exclusion activity demonstrated	Live ZY-312 promoted group 3 innate lymphoid cell (ILC3)-derived interleukin-22 (IL-22) production and IL-22-dependent signal transducer and activator of transcription 3 (STAT3) phosphorylation in the specified model. Separately, purified TP2 was associated with phosphoinositide 3-kinase (PI3K)/protein kinase B (Akt)-related signaling changes and improvements in barrier-associated endpoints in TNBS-induced colitis.	Dextran sulfate sodium (DSS)-induced colitis in mice; TNBS-induced colitis in rats treated with TP2; *C. sakazakii*-induced NEC in neonatal rats
NCTC 9343 (ATCC 25285) [49,50]	Reference/type strain	Not assessed in Ref	PSA dependence was not experimentally evaluated	Not investigated	Inhibited TNF-α-induced nuclear factor kappa B (NF-κB) activation and epithelial injury, promoted epithelial cell migration, and regulated inflammatory cytokines in hcoEPIC cells; live bacteria alleviated DSS-induced colitis by enhancing epithelial barrier integrity, regulating inflammatory cytokines, and modulating the gut microbiota and fecal SCFAs.	TNF-α-induced inflammatory injury in hcoEPIC cells in vitro; DSS-induced acute colitis in C57BL/6J mice
SNBF-1 [21]	Healthy baby faeces	*Bft*-negative by PCR	Not investigated	Not investigated	Cholesterol removal, bile salt hydrolase activity, attenuation of lipid accumulation, modulation of glucose metabolism, and antioxidant activity	Fatty acid-induced and insulin-resistant HepG2 cell models in vitro
BF-1153 [39]	Normal calf feces	*Bft*-negative by PCR and whole-genome analysis	Capsule-associated genes were predicted, but PSA was not specifically characterized	Not investigated	Genome-predicted metabolic and carbohydrate-utilization functions; the cell-free supernatant showed no cytotoxicity and enhanced the viability of HCT-8, MDBK, and IPEC cells	Safety assessment in healthy specific-pathogen-free (SPF) Kunming mice; preventive effects against bovine rotavirus (BRV)-induced diarrhea and small-intestinal injury in SPF Kunming mice
HCK-B3 [35,46]	Feces from a healthy Chinese donor	*Bft* not detected by whole-genome analysis	Four conserved PSA-biosynthesis genes identified (>98% similarity to ATCC 25285); PSA not experimentally confirmed	Putative/incomplete T6SS: *clpV* and *clpB* detected, but VgrG and Hcp absent; function not verified	Reduced TNF-α, increased IL-10, restored Treg cells, and counteracted LPS-associated microbiota disturbances; no evident toxicity in healthy or immunosuppressed mice	LPS-induced inflammation and cyclophosphamide (CTX)-induced immunosuppression models in mice

Abbreviations: NTBF, nontoxigenic *Bacteroides fragilis*; bft, *B. fragilis* toxin gene; PSA, polysaccharide A; TP2, a ZY-312-derived zwitterionic capsular polysaccharide preparation; T6SS, type VI secretion system; NCTC, National Collection of Type Cultures; ATCC, American Type Culture Collection; ILC3, group 3 innate lymphoid cell; IL-22, interleukin-22; STAT3, signal transducer and activator of transcription 3; PI3K, phosphoinositide 3-kinase; Akt, protein kinase B; DSS, dextran sulfate sodium; TNBS, 2,4,6-trinitrobenzene sulfonic acid; NEC, necrotizing enterocolitis; TNF-α, tumor necrosis factor alpha; NF-κB, nuclear factor kappa B; IL-10, interleukin-10; SCFAs, short-chain fatty acids; PCR, polymerase chain reaction; SPF, specific-pathogen-free; BRV, bovine rotavirus; Treg, regulatory T cell; LPS, lipopolysaccharide; CTX, cyclophosphamide; hcoEPIC, human normal colonic epithelial cells; HCT-8, human ileocecal adenocarcinoma cells; MDBK, Madin–Darby bovine kidney cells; IPEC, intestinal porcine epithelial cells; HepG2, human hepatocellular carcinoma cells; *clpV* and *clpB*, genes encoding Clp-family ATPases; VgrG, valine–glycine repeat protein G; Hcp, hemolysin-coregulated protein; *C. sakazakii*, *Cronobacter sakazakii*; kDa, kilodaltons.

**Table 2:** Evidence from representative in vivo NTBF-specific disease models.

Disease/Model	Intervention	Main Evidence	Evidence Level and Limitations
IBD: live ZY-312; DSS-treated mice [16]	1 × 10^9^ CFU/day before dextran sulfate sodium (DSS); PBS and pathway-deficient controls	Reduced colitis severity; interleukin-22 (IL-22) or epithelial signal transducer and activator of transcription 3 (STAT3) disruption attenuated protection, supporting IL-22–STAT3-dependent repair	A. Causal pathway evidence, but limited to one strain, dose, and prophylactic acute chemical model
IBD: purified TP2; TNBS-treated rats [47]	1–4 mg/kg/day for 7 days after TNBS; model and prednisolone controls	Improved disease activity, histology, and barrier-associated endpoints; PI3K/Akt-related effects proposed	B. Purified preparation and short chemical model; no causal pathway intervention; effects not uniformly dose dependent
NEC: live ZY-312; pathogen–hypoxia neonatal rats [48]	1 × 10^9^ CFU/day before challenge; normal and NEC-model controls	Reduced mortality, intestinal injury, permeability, and inflammatory or cell-death-associated markers	B. Prophylactic single-dose neonatal-rat model; limited similarity to heterogeneous human preterm NEC
AAD: live ZY-312; antibiotic-treated rats [54]	10^7^–10^9^ CFU/day for 4–7 days; model and Bifico controls	Improved diarrhea and barrier-associated endpoints; strongest effects at 10^9^ CFU/day	B. Dose-dependent short rat model; incomplete microbiota recovery and no clinical validation
CDI-related AAD: live ZY-312; antibiotic-disrupted mice [40]	10^7^ or 10^8^ CFU/day before *C. difficile* challenge; CDI and metronidazole controls	Improved survival, histology, barrier markers, and community composition; lower dose was not inferior to the higher dose	B. Prophylactic design and non-monotonic dose response; established CDI was not treated
CRC-related model: live NCTC 9343 or ΔPSA mutant; ETBF-challenged mice [73]	Administered before, simultaneously with, or after ETBF; ETBF-alone and timing controls	Reduced colitis and tumors only when administered before ETBF; ΔPSA remained protective	A. PSA-independent but strictly timing-dependent prophylaxis; no efficacy after stable ETBF colonization or established cancer

Category A: in vivo evidence incorporating pathway interruption, knockout or isogenic-strain comparisons, or intervention-timing controls. Category B: in vivo functional-efficacy evidence without complete causal validation. Category C evidence, including cell-line studies and human observational associations, was excluded from this table and is discussed separately in the text. CFU, colony-forming units; DSS, dextran sulfate sodium; TNBS, 2,4,6-trinitrobenzene sulfonic acid; NEC, necrotizing enterocolitis; AAD, antibiotic-associated diarrhea; CDI, *Clostridioides difficile* infection; ETBF, enterotoxigenic *Bacteroides fragilis*; NTBF, nontoxigenic *Bacteroides fragilis*; IBD, inflammatory bowel disease; PBS, phosphate-buffered saline; IL-22, interleukin-22; STAT3, signal transducer and activator of transcription 3; TP2, a ZY-312-derived zwitterionic capsular polysaccharide preparation; PI3K, phosphoinositide 3-kinase; Akt, protein kinase B; CRC, colorectal cancer; NCTC, National Collection of Type Cultures; PSA, polysaccharide A; ΔPSA, PSA-deficient mutant; *C. difficile*, *Clostridioides difficile*.

## Data Availability

No new data were created or analyzed in this study. Data sharing is not applicable to this article.
